# Effect of environmental and pharmaceutical exposures on fetal testis development and function: a systematic review of human experimental data

**DOI:** 10.1093/humupd/dmz004

**Published:** 2019-03-14

**Authors:** Karen R Kilcoyne, Rod T Mitchell

**Affiliations:** 1 MRC Centre for Reproductive Health, The University of Edinburgh, Queen’s Medical Research Institute, 47 Little France Crescent, Edinburgh, UK; 2 Royal Hospital for Sick Children, Edinburgh, UK

**Keywords:** human fetus, testis, endocrine disruptor, environmental chemical, pharmaceutical, testosterone, germ cell, Leydig cell, Sertoli cell, steroidogenesis

## Abstract

**BACKGROUND:**

Overall, the incidence of male reproductive disorders has increased in recent decades. Testicular development during fetal life is crucial for subsequent male reproductive function. Non-genomic factors such as environmental chemicals, pharmaceuticals and lifestyle have been proposed to impact on human fetal testicular development resulting in subsequent effects on male reproductive health. Whilst experimental studies using animal models have provided support for this hypothesis, more recently a number of experimental studies using human tissues and cells have begun to translate these findings to determine direct human relevance.

**OBJECTIVE AND RATIONALE:**

The objective of this systematic review was to provide a comprehensive description of the evidence for effects of prenatal exposure(s) on human fetal testis development and function. We present the effects of environmental, pharmaceutical and lifestyle factors in experimental systems involving exposure of human fetal testis tissues and cells. Comparison is made with existing epidemiological data primarily derived from a recent meta-analysis.

**SEARCH METHODS:**

For identification of experimental studies, PubMed and EMBASE were searched for articles published in English between 01/01/1966 and 13/07/2018 using search terms including ‘endocrine disruptor’, ‘human’, ‘fetal’, ‘testis’, ‘germ cells’, ‘testosterone’ and related search terms. Abstracts were screened for selection of full-text articles for further interrogation. Epidemiological studies involving exposure to the same agents were extracted from a recent systematic review and meta-analysis. Additional studies were identified through screening of bibliographies of full-texts of articles identified through the initial searches.

**OUTCOMES:**

A total of 25 experimental studies and 44 epidemiological studies were included. Consistent effects of analgesic and phthalate exposure on human fetal germ cell development are demonstrated in experimental models, correlating with evidence from epidemiological studies and animal models. Furthermore, analgesic-induced reduction in fetal testosterone production, which predisposes to the development of male reproductive disorders, has been reported in studies involving human tissues, which also supports data from animal and epidemiological studies. However, whilst reduced testosterone production has been demonstrated in animal studies following exposure(s) to a variety of environmental chemicals including phthalates and bisphenol A, these effects are not reproduced in experimental approaches using human fetal testis tissues.

**WIDER IMPLICATIONS:**

Direct experimental evidence for effects of prenatal exposure(s) on human fetal testis development and function exists. However, for many exposures the data is limited. The increasing use of human-relevant models systems in which to determine the effects of environmental exposure(s) (including mixed exposures) on development and function of human tissues should form an important part of the process for assessment of such exposures by regulatory bodies to take account of animal–human differences in susceptibility.

## Introduction

Development of the male reproductive system and its subsequent function is impacted by events that occur *in utero*. Perturbations in testicular development or function during fetal life may result in male reproductive disorders that present postnatally (van den Driesche *et al.*, 2017). This includes anatomical abnormalities identified at birth, such as cryptorchidism and hypospadias, or disorders presenting in adulthood, including testicular cancer or infertility (Sharpe and Skakkebaek, 2008). These associated disorders are collectively referred to as the testicular dysgenesis syndrome (TDS). The development of TDS has been shown in rats to be influenced by a reduction in androgen production or action during a key period of fetal life, known as the masculinization programming window (MPW) (Welsh *et al.*, 2008; van den Driesche *et al.*, 2017). The increasing incidence of TDS disorders over recent decades, highlights the potential importance of environmental impacts in their etiology (Skakkebaek *et al.*, 2016). Environmental factors that have been proposed to affect fetal testis development and predispose to TDS disorders include environmental chemicals (e.g. plasticizers and pesticides), pharmaceuticals (e.g. analgesics, metformin and diethylstilboestrol) and lifestyle factors (e.g. diet, alcohol and smoking) (Habert *et al.*, 2014; Kilcoyne and Mitchell, 2017).

**Figure 1:**
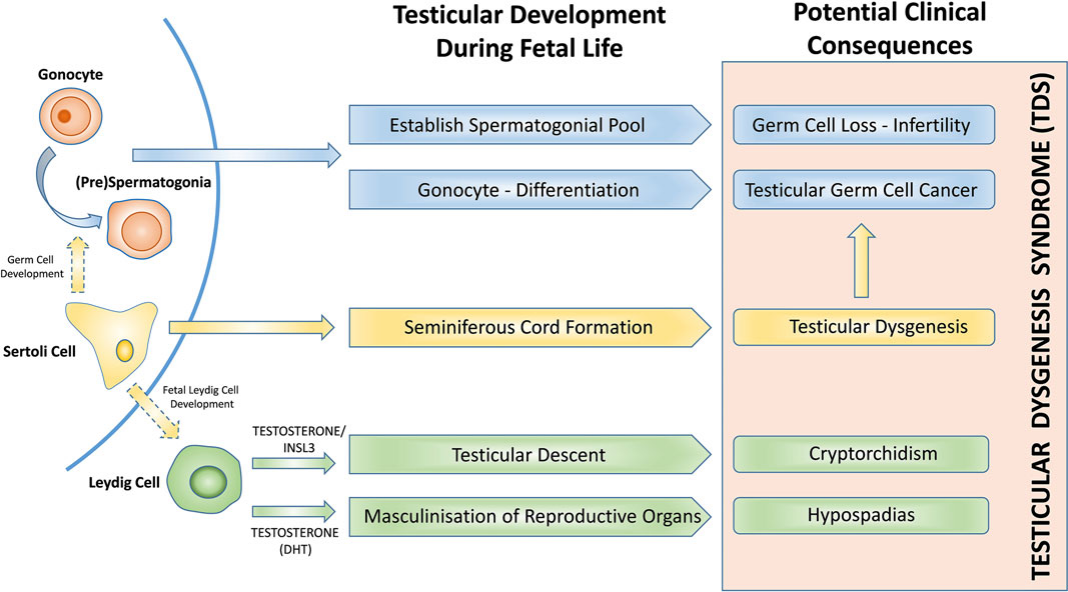
**Testicular development and function during fetal life and reproductive disorders associated with testicular dysgenesis syndrome.** DHT, dihydrotestosterone.

In order to understand how *in-utero* exposures might disrupt fetal development and result in postnatal testicular disorders, it is important to consider the normal development of the germ and somatic cell populations in the human fetal testis (Fig. 1). During fetal life, germ cells migrate into the developing gonad (4–5 weeks in human) where they undergo differentiation from gonocytes to spermatogonia. This transition takes place during fetal and early postnatal life and involves the loss of expression of pluripotency factors (e.g. POU5F1) and gain of differentiated germ cell-specific protein expression (e.g. MAGEA4) (Mitchell *et al.*, 2008). Failure of gonocyte differentiation can result in the development of pre-malignant germ cell neoplasia *in-situ* cells (GCNIS), which results in the development of testicular germ cell cancer (TGCC) in adulthood (Rajpert-De Meyts *et al.*, 2016) whilst loss of germ cells as a result of *in-utero* events can also potentially impact future fertility.

Germ cell development during fetal life is supported by somatic cells that form the germ-stem cell niche. Sertoli cells surround the gonocytes, forming seminiferous cords, at ~6–7 gestational weeks (GW) in the human (O’Shaughnessy and Fowler, 2011; Heeren *et al.*, 2015). Sertoli cells are fundamental for germ cell development (Bitgood *et al.*, 1996), regression of the Müllerian ducts (AMH, anti-Müllerian hormone) and initiation of fetal Leydig cell differentiation (Pierucci-Alves *et al.*, 2001; Yao *et al.*, 2002; Griswold and Behringer, 2009).

Fetal Leydig cells are present in the interstitium of the testis from six GWs in humans and are responsible for the production of hormones involved in testicular descent. Insl3 is involved in the transabdominal phase of testicular descent, whilst testosterone is required to enable the testis to traverse the inguinal canal (Hughes and Acerini, 2008). Leydig cell-derived testosterone is also converted to dihydrotestosterone (DHT) in peripheral tissues for masculinization of the fetus, which includes development of external genitalia. Therefore, perturbations to the function of fetal Leydig cells can predispose to the development of male reproductive disorders including TDS and some disorders of sex development (DSD) (van den Driesche *et al.*, 2017).

The majority of experimental studies investigating the effects of environmental exposures on fetal testis development and function involve rodents. These studies have provided a large amount of valuable information highlighting the potential for effects of in-utero exposure to a wide variety of environmental chemicals on male reproductive development. However, there are important differences in fetal testicular development between rodent and human in terms of germ cell development (McKinnell *et al.*, 2013) and steroidogenesis (Scott *et al.*, 2009). Furthermore, the exposures used for these studies may not reflect the levels of exposure that are directly relevant to humans. Assessment of experimental studies using experimental animal models must take account of the variations in model systems (*in-vitro* versus *in-vivo*), exposure regimen and drug metabolism, whilst also accounting for the impact of species differences for each of these parameters (Kilcoyne and Mitchell, 2017). As a result, animal studies often report findings based on relative exposures which exceed human-relevant exposures, often by several orders of magnitude.

**Figure 2:**
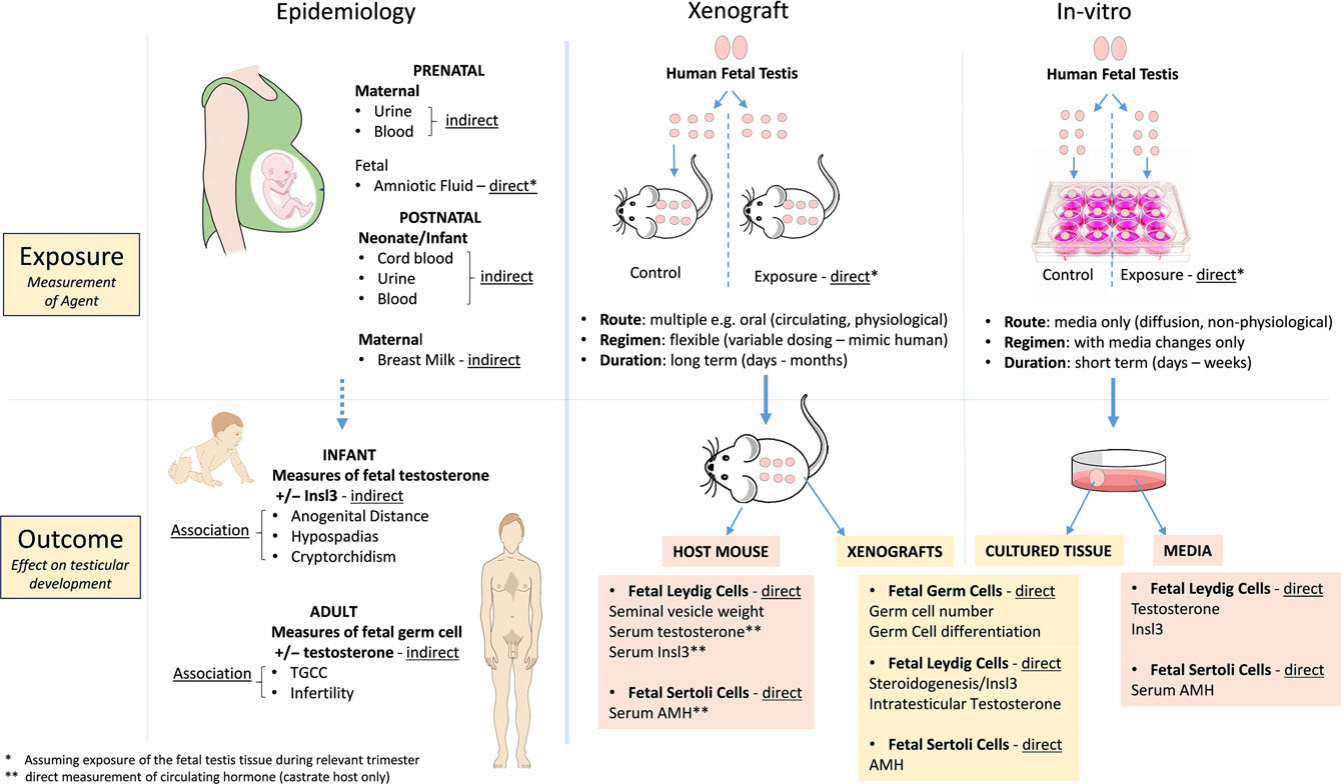
**Comparison of approaches to comparing the effects of environmental and pharmaceutical exposures on human fetal testis development and function.** TGCC, testicular germ cell cancer.

In order to gain information on the potential for *in-utero* environmental exposures to impact development of male reproductive disorders in humans, epidemiological studies can be employed. A recent systematic review has described the epidemiological evidence for associations between prenatal exposures and male reproductive disorders in humans (Bonde *et al.*, 2016). A number of important considerations must be applied when assessing epidemiological evidence for such associations. These include, but are not limited to (i) the relevance and/or size of the population group, (ii) measurement of the exposure (direct/indirect) (iii) biological plausibility for the exposure alone causing the effect and elimination of potential confounders (reviewed in Foster *et al.*, 2017) (Fig. 2 and Table I).

**Table I:** Key considerations for the assessment of human studies using epidemiological, or experimental (xenograft or *in-vitro*) approaches.

	Epidemiology	Xenograft	In vitro
**Population**	Potential limitation—population/tissue used may be unrepresentative of target population/tissue		
	Is the population studied representative of the target population (e.g. pregnant women)?	Is the tissue representative of the target population (e.g. fetal tissue)?	Is the tissue representative of the target population (e.g. fetal tissue or cells)?
**Agent**	Potential limitation—agent under investigation may be not be representative of the exposure under investigation (e.g. metabolite) or there may be additional confounding agents		
	Is the relevant agent being measured in the population? Is there potential for confounding by other/similar exposures?	Is the investigated agent relevant to potential human exposure? Is it metabolized in the host animal to an active/inactive form (e.g. DBP–MBP)?	Is the investigated agent relevant to potential human exposure? Is the active agent or metabolite added to the medium?
**Exposure**	Potential limitation—assessment of exposure in epidemiological studies or regimen used in experimental studies may not accurately reflect true human exposure		
	Is the exposure measured in the subjects (e.g. indirect measurement of fetal exposure through maternal serum/urine) or indirectly assessed (e.g. self-report) and does this accurately capture actual fetal exposure?	Is the dose, frequency, duration and route of exposure? representative of human exposure (e.g. pharmaceuticals)?	Is the concentration of agent placed in the media representative of human levels (e.g. maternal serum, amniotic fluid, fetal serum) and/or human dosing regimen?
**Timing**	Potential limitation—timing of assessment of exposure in epidemiological studies or developmental stage of tissue used in experimental studies may not accurately reflect the relevant stage		
	Is exposure measured at the appropriate developmental stage (e.g. trimester of fetal life or MPW)? Does timing and frequency of measurement accurately reflect internal exposure?	Is the transplanted tissue at the same developmental stage (e.g. trimester of fetal life or MPW)? Does the experimental system maintain tissue development and function?	Is the tissue cultured at the same developmental stage (e.g. trimester of fetal life or MPW)? Does the experimental system maintain tissue development and function?
**Effect**	Potential limitation—the effects of exposure may be measured directly or through association and direct effects of exposure may not result in clinical consequences		
	Is there a direct clinical association with fetal exposure (e.g. cryptorchidism) or is it a surrogate marker for clinical effects (e.g. AGD)? What is the magnitude of effect and is it statistically significant? Is there a plausible mechanism?	Is the effect clinically relevant (e.g. potential for reduced testosterone to induce cryptorchidism)? What is the magnitude of effect and is it statistically significant? Has the mechanism for the effect been defined?	Is the effect relevant to *in-vivo* situation? Is the effect clinically relevant (e.g. potential for reduced testosterone to induce cryptorchidism)? What is the magnitude of effect and is it statistically significant? Has the mechanism for the effect been defined?

While human epidemiological and animal experimental studies are extremely informative, there remains a large gap in our understanding of how specific environmental exposures may directly affect the human fetal testis. Therefore, development of model systems using human fetal tissues and human-relevant doses can bridge the gap between direct evidence from animal experimental models and indirect evidence based on epidemiological data. A number of recent studies have utilized *in-vitro* or xenograft approaches using human fetal testis tissues to determine the effect of environmental and pharmaceutical exposures. As with epidemiological studies, there are a number of key considerations when interpreting the results of these studies relating to model system, exposure regimen and biological relevance of the measured outcome (Fig. 2 and Table I).

A comprehensive review of the experimental evidence for effects of environmental exposures on fetal testicular development and function using human cells or tissues has not previously been reported. This systematic review will detail the experimental evidence for impacts of environmental chemicals, pharmaceuticals and lifestyle factors on human fetal testis development and function. For each class of exposure for which human experimental evidence exists, we will first summarize the findings of animal studies and then provide a critical review of the epidemiological evidence. We will then describe in detail the evidence to support or refute these findings based on experimental models using human fetal testis tissues.

## Methods

The study was designed as a systematic review of the published literature relating to the effects of *in-utero* exposures on human fetal testis development and function in experimental models. The study followed the principles of the PRISMA guidelines for reporting systematic reviews (Moher *et al.*, 2009). The protocol for searching and assessing the literature was determined prior to the start of the literature search. It is not currently possible to register laboratory experimental studies with PROSPERO.

### Information sources

We performed an online search of PubMed and EMBASE (13/07/2018) to identify all experimental studies relating to testicular effects of fetal exposures to environmental, pharmaceutical and lifestyle factors, limited to studies utilizing human fetal tissues or cells. For identification of relevant epidemiological studies, we included the publications identified in a recent systematic review and meta-analysis of associations between prenatal exposures and male reproductive disorders (Bonde *et al.*, 2016). Additional epidemiological studies were identified from the reference lists of the screened articles.

### Eligibility for inclusion

We performed a systematic search of original publications according to the following criteria for inclusion: English language articles published between 01/01/1966 and 13/07/2018; experimental studies on exposure of human fetal testis tissue or cells to a clearly defined environmental, pharmaceutical or lifestyle factor; and outcomes including effects on testicular hormone production (e.g. testosterone, Insl3, AMH), germ or somatic (Sertoli, Leydig) cell development.

### Exclusion criteria

We excluded studies according to the following criteria: exposure of tissues or cells representative of a period other than fetal life; exposure of non-testicular tissues or cells; outcomes other than those described above; and review articles.

### Search and study selection

**Figure 3:**
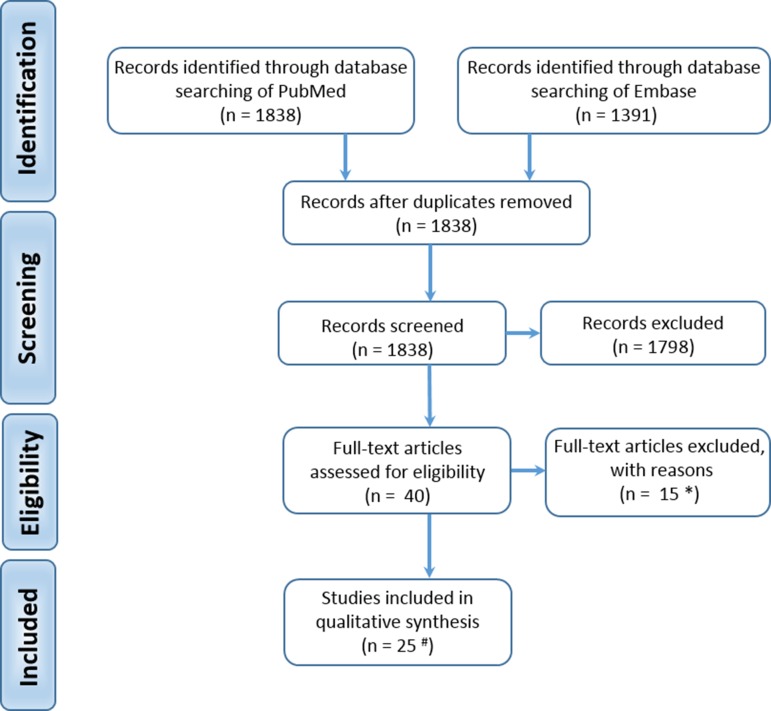
**Prisma flow diagram for identification and selection of studies.** ^#^ Supplementary Table S2; * Supplementary Table S3.

We searched the databases using a combination of medical subject headings and generic terms relating to effects of exposures on human fetal testis development (Supplementary Table S1). We identified 3229 hits. Both authors screened the titles and/or abstracts independently to assess eligibility. Full texts were requested for studies that included in the abstract the use of human fetal testis tissue or cells and the effect of exposure to environmental, pharmaceutical or lifestyle factors. Full texts for 40 studies were obtained and a total of 25 publications were included in the review of experimental evidence (Supplementary Table S2; Fig. 3). A total of 15 studies were excluded (Supplementary Table S3). A further 44 publications were included in the review of epidemiological evidence (Supplementary Table S4).

### Summary measures

Studies included *in-vitro* and *ex-vivo* (xenograft) approaches and results were assessed primarily for effects on testosterone secretion and on germ cell number (both expressed as % change compared to vehicle control). Effects on additional testicular hormones, AMH (produced by SC) and Insl3 (produced by LC) are also reported.

## Results

**Figure 4:**
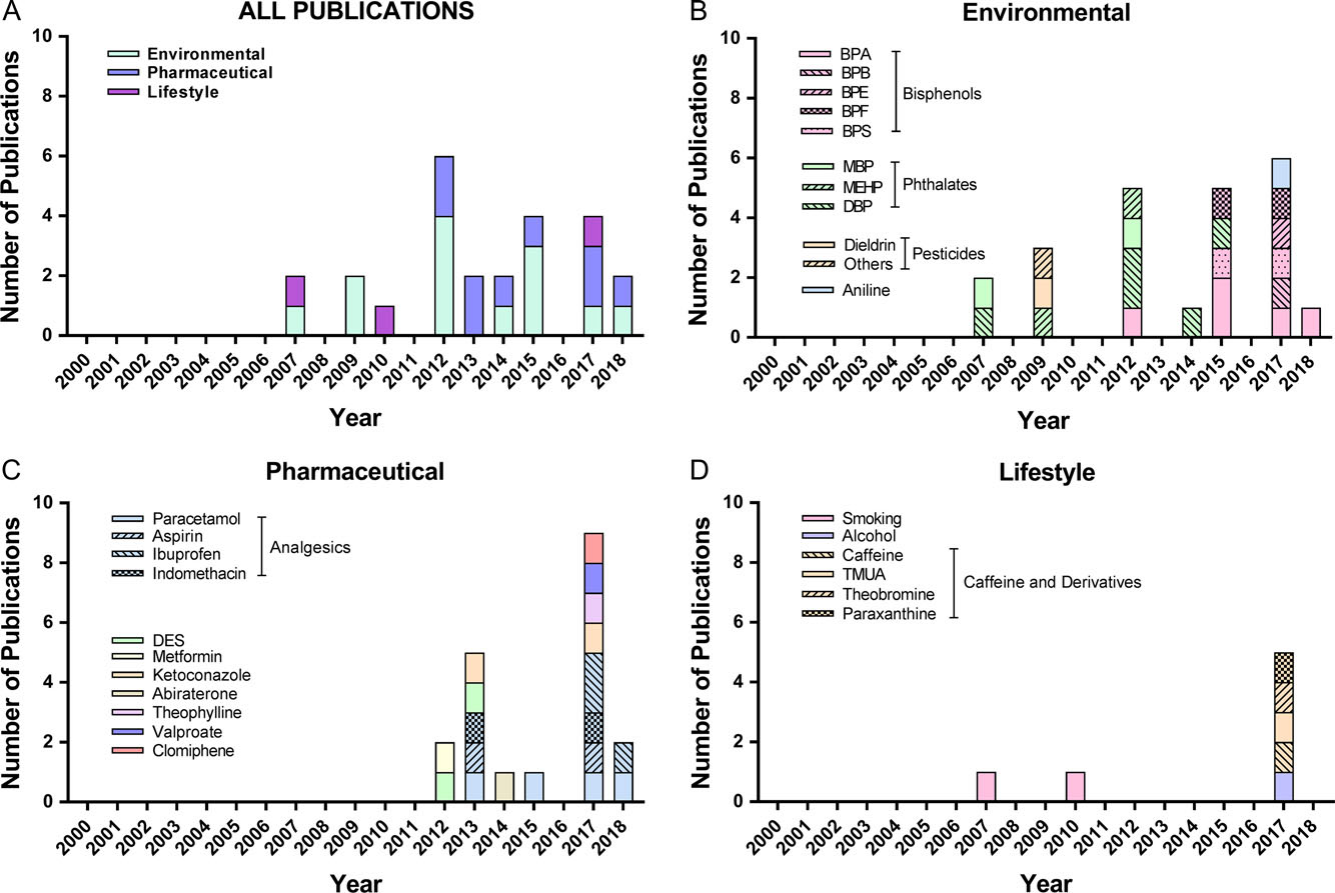
**Number of publications involving experimental exposures to environmental agents and pharmaceuticals using human fetal testis tissues or cells.** DES, diethylstilboestrol. For (**A**) ‘All Publications’, a breakdown of the investigated agents into (**B**) environmental, (**C**) pharmaceutical and (**D**) lifestyle is included. NB: Some publications include exposure to several different agents.

The distribution of studies based on exposure type and year of publication is shown in Fig. 4. The majority of the studies were published from 2007 to 2018. The earlier studies primarily investigated phthalates, pesticides and smoking, whilst more recent studies have mainly focused on bisphenols and analgesics.

## Environmental chemicals

### Phthalates

Phthalates are a class of industrial chemicals used mainly to soften polyvinyl chloride-based products and are found in a wide array of general plastic products. Exposure to phthalates may occur via inhalation, ingestion or direct contact with items including packaging, oils, food storage and personal care products. Phthalates are not stored in the body but are instead rapidly metabolized into monoesters with a urinary excretion half-life of <24 h (Koch *et al.*, 2012). Commonly used plasticisers over recent decades include di-(2-ethylhexyl) phthalate (DEHP) which is converted to its monoester metabolite, mono-2-ethylhexyl phthalate (MEHP) and di-*n*-butyl phthalate (DBP), which is metabolized to mono-*n*-butyl phthalate MBP. In 2015, DEHP was banned from general use under EU law under the REACH restriction (Registration, Evaluation, Authorization and Restriction of Chemical Substances) and has been replaced by di-isononyl phthalate (DiNP), and more recently di-isononyl cyclohexane-1,2-dicarboxylate (DINCH), both of which are deemed less biologically active (Gray *et al.*, 2000). Recent biomonitoring data has shown that DiNP metabolites measured in human urinary samples are increasing in both America (Silva *et al.*, 2013) and Europe (Goen *et al.*, 2011).

#### Animal studies

The majority of published experimental studies investigating the effects of *in-utero* exposure to certain phthalates (primarily DEHP or DBP) have used rodent models and have demonstrated a disruption of normal fetal testis development and subsequent development of male reproductive disorders, resulting from decreased fetal testosterone production in male offspring (Parks *et al.*, 2000; Mylchreest *et al.*, 2002; Habert *et al.*, 2014; Kilcoyne *et al.*, 2014; Fisher *et al.*, 2016). Moreover, effects primarily result from exposure during the MPW (Welsh *et al.*, 2008; Kilcoyne *et al.*, 2014; van den Driesche *et al.*, 2017) which is believed to equate to ~8–14 weeks gestation in humans (Scott *et al.*, 2009; Fowler *et al.*, 2011).

#### Epidemiology - DBP/MBP

In humans, DBP is rapidly metabolized (primarily to MBP) and excreted (Koch *et al.*, 2012). Therefore measuring the effects of phthalate exposure on fetuses in pregnant women is challenging. Despite this, urinary concentrations of several phthalate metabolites (including MBP) have been shown to be positively correlated with the incidence of male developmental abnormalities in the newborn, such as cryptorchidism and shortened anogenital distance (AGD; a biological readout of fetal testosterone exposure) (Marsee *et al.*, 2006). Further indirect evidence for effects of maternal MBP/DBP exposure on steroidogenesis in the human fetal testis has also been reported in retrospective association studies (Marsee *et al.*, 2006; Swan, 2008). These studies demonstrate a negative correlation between maternal urinary MBP concentration and AGD in males, although the changes are minor. However, a separate small prospective study failed to find any correlation (Huang *et al.*, 2009). Furthermore, whilst an association between urinary MBP during second and third trimester and AGD has been demonstrated (Swan *et al.*, 2005), a similar study has shown no such association when urinary MBP is measured during the first trimester (Swan *et al.*, 2015).

Several phthalate monoesters have been detected in maternal breast milk over a large concentration range (1.5–1 410 μg/L) and these have been correlated with infant hormone levels (Main *et al.*, 2006). Despite the reported association between phthalates (including MBP) in maternal breast milk and altered testosterone levels of infant males (Main *et al.*, 2006), a recent study did not detect significant associations between MBP concentrations in breast milk and either testosterone or INSL3 in newborns (Chevalier *et al.*, 2015). Importantly, this study found no association between MBP in breast milk and the presence of cryptorchidism, consistent with maternal MBP concentrations postnatally not being associated with human fetal testosterone production.

Phthalates, including DBP, have also been used in the enteric coating of several commonly used medications including proton pump inhibitors (e.g. omeprazole) and anti-epileptics (e.g. valproate, carbemazepine) (Kelley *et al.*, 2012; Jamieson and McCully, 2015). This can result in an increase in urinary phthalate levels in the region of 50–100 times, compared with the general population (Hernandez-Diaz *et al.*, 2009; Seckin *et al.*, 2009; Hait *et al.*, 2014). As a result, it may be expected that the regular use of these medications during pregnancy may be associated with an increased risk of male reproductive disorders in the offspring of exposed mothers. DBP has been used in the coating of mesalazine (a 5-aminosalicylic acid drug), an anti-inflammatory agent used to treat inflammatory bowel disease (IBD). Several studies have reported the pregnancy outcomes for women taking 5-ASA drugs during pregnancy and a meta-analysis of 2200 pregnant women with IBD has reported no increased incidence of congenital abnormalities as a result of exposure to mesalazine during pregnancy (*n* = 642), in comparison to non-exposed mothers with IBD (*n* = 1158) (Rahimi *et al.*, 2008). It is important to point out that a limitation of the studies included in the meta-analysis is that they were not specifically designed to identify associations with male reproductive disorders; however, it is reassuring to note that none of the studies to date have reported an increase in the development of these disorders. Prospective studies designed to answer this specific question would be desirable, although conducting such studies will be challenging due to the widespread removal of DBP from these medications.

#### Epidemiology - DEHP/MEHP

DEHP has been detected in fetal cord blood samples (of 1.1 × 10^−8^ M) and cord blood of newborns (Latini *et al.*, 2003; Lin *et al.*, 2011), demonstrating that fetal exposure to DEHP can begin from an early stage. MEHP, measured in the urine of pregnant women (*n* = 111; 9–40 weeks gestation) has been associated with a significant reduction in AGD in male offspring (Suzuki *et al.*, 2012). DEHP metabolites in pregnant women during the first trimester have been shown to be significantly and inversely related with AGD in male newborns (*n* = 366) (Swan *et al.*, 2015), whereas no such association was identified for maternal urinary MEHP in second or third trimesters (Swan *et al.*, 2005). A similar inverse relationship between the anoscrotal distance (AGDas) in newborn males (*n* = 168) and maternal DEHP metabolite, concentrations specifically during the first trimester, has also been described (Martino-Andrade *et al.*, 2016). These findings are in contrast to a Swedish study which found no significant association between first trimester concentrations of DEHP metabolites in mothers and AGDas in 196 males aged 21 months (Bornehag *et al.*, 2015). Interestingly, this is despite the authors reporting a significant association between AGDas (4% reduction) and some DiNP metabolites (Bornehag *et al.*, 2015).

The timing of measurement of exposure may be important given that whilst an association between first trimester maternal urine concentrations of DEHP and AGDas have been described, no association was found for second or third trimester DEHP concentrations (Martino-Andrade *et al.*, 2016). Another recent study measured urinary phthalates (including DEHP metabolites) concentration in pregnant women during late second and third trimester and reported no association between prenatal phthalate exposure and AGD in male offspring (*n* = 273) at 3 months of age (Jensen *et al.*, 2016). Furthermore, a study involving measurement of 5cx-MEPP (a DEHP metabolite) in amniotic fluid during the second trimester found no association with cryptorchidism or hypospadias (Jensen *et al.*, 2015).

#### Experimental evidence from human studies

Investigating phthalate exposure directly using human fetal testis is challenging; however, several studies have utilized *in-vitro* cell or tissue culture and/or *in-vivo* xenograft models to explore potential human effects (Table II).

**Table II:** Summary of experimental studies investigating effects of phthalate exposure in human fetal testis tissue.

Model and regimen							Results					
Exposure	Model	Fetal age (weeks)	Dose	Route	Regimen	Supplemented	Testosterone	AMH	INSL3	Germ cells	Study	Comments
DBP	Xenograft	14–20	500 mg/kg/d	Oral	4 or 21 days	hCG				↓Gonocytes, ↑MNG	van den Dreische (2015b)	
	Xenograft	14–20	500 mg/kg/d	Oral	4 or 21 days	hCG	↔			↔	Mitchell *et al.* (2012)	
	Xenograft	16–22	500 mg/kg/d	Oral	14 days	hCG	↔			↔	Spade *et al.* (2014)	
	Xenograft	10–24	100–500 mg/kg/d	Oral	24–72 h	Nil			↔	↑MNG	Heger *et al.* (2012)	
	*In-vitro*	15-20	10^−3^ M	Media	24-48 h	Basal/hCG/22 R-OH	↔				Hallmark *et al.* (2007)	
MBP	Xenograft	14–20	500 mg/kg/d	Oral	4 or 21 days	hCG	↔				Mitchell *et al.* (2012)	
	*In-vitro*	15–20	10^−3^ M	Media	24–48 h	Basal/hCG/22 R-OH	↔				Hallmark *et al.* (2007)	
MEHP	*In-vitro*	7–12	10^−4^ M	Media	72 h	LH	↔	↔		↓GC (40%)	Lambrot *et al.* (2009)	
	*In-vitro*	7–12	10^−5^ M	Media	72 h	LH	↔		↔	↔GC number	Lambrot *et al.* (2009)	
	*In-vitro*	7–12	10^−6^ M	Media	72 h	LH	↔	↔	↔	↔GC number	Lambrot *et al.* (2009)	
	*In-vitro*	7–12	10^−5^ M	Media	72 h	Nil				↑Apoptosis (~45%)*	Muczynski and Cravedi *et al.* (2012)	*Gonocytes

Significant effects associated with adverse outcomes are highlighted in red, no change or significant effects not expected to result in adverse outcomes are highlighted in green.

MNG, multinuclear gonocytes; GC, germ cells; 22 R-HO, 22 R-hydroxycholesterol; SV, seminal vesicle.

##### DBP/MBP - Hormones

Effects of exposure to DBP/MBP have been investigated in human fetal testis tissues using *in-vitro* or xenograft approaches. *In-vitro* exposure of second trimester human fetal testis explants to MBP had no effect on (basal or hCG stimulated) testosterone production after 48 h (Hallmark *et al.*, 2007), whilst the same study demonstrated a reduction in testosterone production in fetal rat testis explants after hCG stimulation (Hallmark *et al.*, 2007). Use of an *ex-vivo* xenograft system may represent a more physiological approach to investigating potential effects of phthalates on the human fetal testis. Despite several studies xenografting first and/or second trimester human fetal testes into different host species, and at different locations (rat; renal subcapsular space) (Heger *et al.*, 2012; Spade *et al.*, 2014) (mouse; subcutaneous tissue) (Mitchell *et al.*, 2012), no effects on steroidogenic gene expression, seminal vesicle weight or circulating testosterone levels were identified at levels of exposure equivalent to those used in rat pregnancy studies, which are far in excess of human exposure. This included varying duration of DBP exposure for 4 or 21 days (Mitchell *et al.*, 2012) or for 14 days (Heger *et al.*, 2012; Spade *et al.*, 2014). Furthermore, substituting MBP instead of DBP for 21 days also had no effect on testosterone production from xenografts (Mitchell *et al.*, 2012). Exposure of human fetal testis xenografts to DBP at varying doses (100, 250, 500 mg/kg) over a more limited time-window (24, 48, 72 h) did not alter mRNA expression of Leydig cell genes including several steroidogenic genes and the hormone Insl3 (produced by Leydig cells and involved in testicular descent) (Heger *et al.*, 2012).

Further support for the lack of effect of exposure to DBP/MBP on testosterone production and development of male reproductive disorders in primates is evident following *in-utero* exposure of marmoset monkeys to MBP (McKinnell *et al.*, 2009). Pregnant marmosets (*n* = 5–6) were dosed with 500 mg/kg MBP from ~7 to 15 weeks gestation and testicular and male reproductive effects were assessed in the neonate or adult. No effects on testicular morphology or germ cell number were identified at birth or adulthood following *in-utero* MBP exposure and testosterone levels at birth were unaffected. Importantly, there were no cases of hypospadias, cryptorchidism, impaired spermatogenesis or focal testicular dysgenesis in any of the exposed animals (McKinnell *et al.*, 2009).

##### DBP/MBP - Germ cells

Despite the lack of effect of DBP exposure on testosterone production or steroidogenic gene expression in the human fetal testis, abnormal morphology of germ cells within the seminiferous cords has been described following DBP exposure in the xenograft model, including an increase in the number of multi-nucleated gonocytes (MNGs) (Heger *et al.*, 2012). Furthermore, DBP-exposure (500 mg/kg) for 21 days in a similar xenograft model reduced the number of gonocytes and induced a higher proportion of MNGs in human fetal testes (van den Driesche *et al.*, 2015b). These effects of DBP exposure, namely reduced gonocyte number and increased MNGs, is in keeping with findings from rodent studies using comparable DBP exposure (Ferrara *et al.*, 2006; Jobling *et al.*, 2011; van den Driesche *et al.*, 2015b).

##### DEHP/MEHP - Hormones

*In-vitro* exposure of human first trimester testis to a range of doses of MEHP (10^−4^, 10^−5^, 10^−6^ M) using an organotypic culture system resulted in unchanged testosterone (basal or LH-stimulated) production compared to control after 72 h of culture (Lambrot *et al.*, 2009). In keeping with a lack of effect on Leydig cell function, there was also no change in gene expression of steroidogenic enzymes or Insl3 (Lambrot *et al.*, 2009). These results are in keeping with similar *in-vitro* (Hallmark *et al.*, 2007) and xenograft (Heger *et al.*, 2012; Mitchell *et al.*, 2012; Spade *et al.*, 2014) studies involving DBP/MBP exposure of human fetal testis tissue.

In addition to assessing effects of MEHP exposure on testosterone production in the human fetal testis, exposure of first trimester testis (7–12 weeks gestation) to 10^−4^ M MEHP for 72 h resulted in a reduction in AMH mRNA, although Sertoli cell-derived AMH protein expression was not affected (Lambrot *et al.*, 2009).

##### DEHP/MEHP - Germ cells

Exposure of human first trimester testis tissue to MEHP (10^−5^ M) *in-vitro* via an organotypic culture system resulted in an approximately 40-50% increase in apoptotic gonocytes compared to control testis tissue (Muczynski and Cravedi *et al.*, 2012). *In-vitro* studies using mouse fetal testis tissue conducted in parallel, demonstrated similar effects with a 30% reduction in gonocyte number and a 4-fold increase in the rate of apoptotic gonocytes (Muczynski and Lecureuil *et al.*, 2012). These results support the findings of a previous study in first trimester human fetal testes exposed to a range of doses of MEHP (10^−4^, 10^−5^, 10^−6^ M) for 72 h. Using a similar *in-vitro* system, MEHP-exposure resulted in a 40% reduction in germ cell number via increased apoptosis (without altering GC proliferation) at both 10^−4^ and 10^−5^ M MEHP under basal conditions, whilst 10^−5^ M MEHP resulted in a reduction in gonocyte number under basal and LH-stimulated conditions (Lambrot *et al.*, 2009). As described for DBP/MBP, the germ cell effects were similar between MEHP-exposed human and mouse fetal testes, as gonocyte number was also significantly reduced in mouse fetal testes after MEHP exposure, via an increase in apoptotic gonocytes (Chauvigne *et al.*, 2009; Lehraiki *et al.*, 2009). However, *in-vitro* organ culture of fetal rat testes (at embryonic Day 13 or 18) with MEHP showed no effect on the mitotically quiescent germ cells (Li and Kim, 2003).

#### Summary—phthalates

Exposure to DBP/DEHP has consistently been shown to reduce fetal testosterone production resulting in a high frequency of TDS disorders in rats, whereas epidemiological studies have reported inconsistent associations between maternal phthalate exposures and indirect measures of fetal testosterone production, namely AGD, and no studies have reported an association between phthalate exposure and either cryoptorchidism or hypospadias. In addition, no effects of phthalates on testosterone production have been described in experimental models using *in-vitro* culture or xenografting of human fetal testis tissues and a single *in-vivo* study in a non-human primate. This is despite administration of phthalate doses that far exceed environmental/human exposure levels and includes exposure during the proposed MPW. Furthermore, the *in-vivo* study involving *in-utero* exposure of marmoset monkeys failed to demonstrate the development of male TDS disorders that result from a reduction in testosterone. Taken together the results indicate that exposure to environmental levels of DBP/DEHP are unlikely to result in effects on fetal testosterone production in humans. Interestingly, whilst no effects of phthalate exposure have been demonstrated in human fetal testes, anti-androgenic effects have been shown to occur in adult human testis following *in-vitro* culture, suggesting that the effect of exposure is dependent on the developmental stage of the testis (Albert and Jegou, 2014).

However, for germ cells effects, the results of experimental studies in rodents and human fetal tissues are more consistent, with a reduction in gonocyte number and an increase in multi-nucleated gonocytes being reported in several studies following phthalate exposure. The implications of these findings in terms of future fertility are uncertain.

### Bisphenols

Bisphenols are synthetic chemicals, widely used in the manufacture of hard plastic products. Bisphenol A (BPA) is also a component of epoxy-resins, used as the inner coating of metallic food and beverage cans. BPA has weak estrogenic properties, albeit several orders of magnitude less potent than endogenous 17β-oestradiol (Leffers *et al.*, 2001).

#### Animal studies

*In-vivo* rodent studies have reported conflicting results regarding the effect of in-utero BPA exposure on testosterone production by the fetal testis. Exposure of pregnant rats to high concentrations of BPA throughout pregnancy was associated with a reduction in testosterone production in male fetuses around birth (Tanaka *et al.*, 2006), whilst other studies have reported no effect of in-utero exposure to BPA on AGD in male offspring (Kobayashi *et al.*, 2002; Howdeshell *et al.*, 2008). *In-vitro* studies have demonstrated a reduction in testosterone production in mouse and rat fetal testis following exposure to high concentrations of BPA (N’Tumba-Byn *et al.*, 2012). Given the conflicting results of animal studies involving BPA, a large collaborative study (CLARITY-BPA) is currently being undertaken in rodents to determine the effects of BPA on a range of body systems including reproductive organs (Schug *et al.*, 2013). A recent publication from the consortium reported no effects of combined *in-utero* and postnatal exposure on several testicular morphometric and histological endpoints, including adult testis weight, except at very high doses that far exceed environmental exposure (Dere *et al.*, 2018).

#### Epidemiology

A limited number of epidemiological studies have investigated the relationship between human pregnancy exposure to BPA and male reproductive disorders. In a study involving measurement of cord blood in 52 newborn boys with cryptorchidism and 126 controls, no association was found between BPA exposure and testosterone; however, a significant negative correlation between BPA and Insl3 was identified (Chevalier *et al.*, 2015). Given that Insl3 and testosterone are both involved in testicular descent, it is interesting to note that there was no association between BPA levels in cord blood and cryptorchidism (Fenichel *et al.*, 2012; Chevalier *et al.*, 2015). Similarly, in another case-control study, no association was identified between BPA exposure, as measured in maternal urine, and cryptorchidism in the male offspring (Chevrier *et al.*, 2012). The relationship between BPA and cryptorchidism has also been investigated in 98 boys with unilateral cryptorchidism aged 1–4 years. Serum BPA was measured prior to surgery and a significant association was found between total BPA and cryptorchidism in boys compared to 57 healthy controls (Komarowska *et al.*, 2015). However, no association was found between free (unconjugated) BPA and cryptorchidism and it is of interest to note the wide variation and high degree of overlap between the BPA levels of the two groups. An association between urogenital abnormality (cryptorchidism or hypospadias) and free BPA levels in term placenta was also described in a study involving 79 boys (Fernandez *et al.*, 2016). In this study, placental BPA was significantly higher in the cases compared to controls; however, an association between BPA and cryptorchidism/hypospadias was only apparent for the upper tertile of BPA exposure which included just 26 (12 cases and 14 controls) boys (Fernandez *et al.*, 2016). In addition, this study was not able to assess BPA exposure during fetal life which temporally separates the measurement of the exposure from the development of the disorder. An association has been described between maternal occupational exposure to BPA and AGD in 56 exposed male offspring when compared to 97 unexposed controls (Miao *et al.*, 2011). Care must be taken when interpreting this data as the exposure to BPA was determined by personal air sampling with extrapolation to estimate past exposure for the index pregnancy.

#### Experimental evidence from human studies

Several experimental studies have investigated the effect of BPA exposure using human fetal testis tissue and cells (Table III). A key consideration when assessing the results of experimental studies involving effects of exposure(s) on the human fetal testis is the concentration/doses to which the tissue is exposed. For BPA, human internal exposure to unconjugated BPA has been reported to be in the range 10^−2^ M to 10^−3^ M, including in pregnant women (Vandenberg *et al.*, 2010) and mean cord blood levels have been reported closer to 10^−3^ M (Fenichel *et al.*, 2012). However, a recent study involving 48 samples from 30 healthy pregnant women, which accounted for potential post-sampling contamination, reported serum BPA concentrations below the LOD apart from 12 (25%) samples which ranged from 10^−9^ to 10^−12^ M, demonstrating that exposures may be substantially lower than previously reported (Teeguarden *et al.*, 2016).

**Table III:** Summary of experimental studies investigating effects of bisphenol exposure in human fetal testis tissue.

Model and regimen							Results					
Exposure	Model	Fetal age (weeks)	Dose	Route	Regimen	Supplemented	Testosterone	AMH	Insl3	Germ cells	Study	Comments
BPA	*In-vitro*	6–11	10^−5^ M	Media	24–72 h	LH	↓ (40–50%)				Eladak *et al.* (2015)	
	*In-vitro*	6–11	10^−8^ to 10^−5^ M	Media	24–72 h	Nil	↓ (20–50%)*				Eladak *et al.* (2015)	*(10^−8^ not significant at 24 h)
	*In-vitro*	6–11	10^−8^ to 10^−5^ M	Media	24–72 h	LH	↔				Eladak *et al.* (2015)	
	*In-vitro*	6–11	10^−8^ to 10^−5^ M	Media	24–72 h	Nil	↓ (20–50%)				N-Tumba Byn (2012)	
	*In-vitro*	6–11	10^−12^ M	Media	24–72 h	Nil	↔				N-Tumba Byn (2012)	
	*In-vitro*	6–12	10^−5^ M	Media	72 h	LH				↓	Eladak *et al.* (2018)	70% ↑ apoptotic gonocytes
	*In-vitro*	6–12	10^−8^ to 10^−6^ M	Media	72 h	LH				↔	Eladak *et al.* (2018)	
	*In-vitro*	7–12	10^−5^ M	Media	72 h	Nil	↓ (70%)	↔	↓		Ben Mamaar (2015)	
	*In-vitro*	7–12	10^−5^ M	Media	72 h	hCG	↓ (60%)	↔	↔		Ben Mamaar (2015)	
	*In-vitro*	7–12	10^−5^ M	Media	72 h	LH	↓ (30%)	↔	↔		Ben Mamaar (2015)	
	*In-vitro*	7–12	10^−8^ to 10^−6^ M	Media	72h	LH	↔	↔	↔		Ben Mamaar (2015)	
	*In-vitro*	7–12	10^−8^ to 10^−6^ M	Media	72 h	hCG	↔	↔	↔		Ben Mamaar (2015)	
	*In-vitro*	7–12	10^−8^ to 10^−6^ M	Media	72 h	Nil	↔**	↔	↔***		Ben Mamaar (2015)	**30% ↓ (10^−8^), ***70% ↓ (10^−8^) and 60% ↓ (10^−5^)
	*In-vitro*	10–12	10^−5^ M	Media	72 h	hCG	↓ (60%)				Gaudriault *et al.* (2017)	
	*In-vitro*	10–12	10^−6^ M	Media	72 h	hCG	↔				Gaudriault *et al.* (2017)	
	Xenograft	9–11	10^−5^ M	Drinking Water	5 weeks	hCG	↔			↓ (19%)^#^	Eladak *et al.* (2018)	^#^19% ↓ gonocytes (↑spermatogonia)
	Xenograft	14–18	0.5, 50 ug/kg	Oral Gavage	5 weeks	hCG	↔				Eladak *et al.* (2018)	
BPB	*In-vitro*	10–12	10^−5^ to 10^−4^ M	Media	72 h	hCG	↓ (25–90%)				Gaudriault *et al.* (2017)	
	*In-vitro*	10–12	10^−8^ to 10^−6^ M	Media	72 h	hCG	↔				Gaudriault *et al.* (2017)	
BPE	*In-vitro*	10–12	10^−4^ M	Media	72 h	hCG	↓ (80%)				Gaudriault *et al.* (2017)	
	*In-vitro*	10–12	10^−7^ to 10^−5^ M	Media	72 h	hCG	↔				Gaudriault *et al.* (2017)	
BPF	*In-vitro*	6–11	10^−8^ M	Media	72 h	Nil	↔				Eladak *et al.* (2015)	
	*In-vitro*	6–11	10^−6^ to 10^−5^ M	Media	72 h	Nil	↓ (30–40%)				Eladak *et al.* (2015)	
	*In-vitro*	10–12	10^−4^ M	Media	72 h	hCG	↓ (60%)				Gaudriault *et al.* (2017)	
	*In-vitro*	10–12	10^−8^ to 10^−5^ M	Media	72 h	hCG	↔				Gaudriault *et al.* (2017)	
BPS	*In-vitro*	6–11	10^−8^ to 10^−7^ M	Media	72 h	Nil	↔				Eladak *et al.* (2015)	
	*In-vitro*	6–11	10^−6^ to 10^−5^ M	Media	72 h	Nil	↓ (50%)				Eladak *et al.* (2015)	
	*In-vitro*	10–12	10^−5^ to 10^−4^ M	Media	72 h	hCG	↓(20–85%)				Gaudriault *et al.* (2017)	
	*In-vitro*	10–12	10^−8^ to 10^−6^ M	Media	72 h	hCG	↔				Gaudriault *et al.* (2017)	

Significant effects associated with adverse outcomes are highlighted in red, no change or significant effects not expected to result in adverse outcomes are highlighted in green.

##### Hormones

A number of studies have investigated the effect of exposure to BPA on testosterone production by human fetal testis tissue using an *in-vitro* system (N’Tumba-Byn *et al.*, 2012; Ben Maamar *et al.*, 2015; Eladak *et al.*, 2015). Exposure of human fetal testis explants (6–11 GW) to BPA did not affect testosterone production at a concentration of 10^−12^ M; however, exposure to higher concentrations (10^−8^ to 10^−5^ M) resulted in a reduction of 20–50% (N’Tumba-Byn *et al.*, 2012). Further studies have compared the effect of BPA exposure under basal and gonadotrophin supplemented conditions (Ben Maamar *et al.*, 2015; Eladak *et al.*, 2015). Exposure of human fetal testis explants (7–12 GW) to 10^−5^ M BPA for 72 h resulted in a significant reduction in testosterone production under basal (70%), hLH- (30%) and hCG-stimulated (60%) conditions (Ben Maamar *et al.*, 2015; Gaudriault *et al.*, 2017). However, exposure to lower concentrations did not result in significant differences except for 10^−8^ M under basal conditions in which a 30% reduction was reported (Ben Maamar *et al.*, 2015). Similar results were obtained in another study in which exposure to BPA for 72 h under basal conditions resulted in a significant reduction in testosterone production by human fetal testis tissue (6–11 GW) across a range of concentrations (10^−8^ to 10^−5^ M), whereas reduction in testosterone only occurred at the highest concentration (10^−5^ M) under hLH supplemented conditions (Eladak *et al.*, 2015). Two separate studies using the xenograft system have also investigated the effect of BPA on testosterone production using first and second trimester human fetal testis tissue (Eladak *et al.*, 2015; Eladak *et al.*, 2018). Exposure of host mice to 10^−5^ M BPA in drinking water for 5 weeks did not affect testosterone production from xenografted tissue, as measured by host mouse seminal vesicle weight or serum testosterone. Importantly, the authors were able to demonstrate that the plasma levels of unconjugated and total BPA were significantly higher in the BPA exposed host mice compared to the vehicle controls. Similarly, for second trimester xenografts, daily oral gavage of host mice with 0.5 or 50 μg/kg BPA for 5 weeks did not impact on testosterone production (Eladak *et al.*, 2018). In both xenograft studies, hCG was administered to the host mice to mimic the hormonal environment of pregnancy as previously described (Mitchell *et al.*, 2010).

The use of gonadotrophin supplementation in the *in-vitro* and *ex-vivo* systems is a key consideration. Supplementation of media with hCG was able to maintain testosterone levels across the culture period, as opposed to hLH or basal conditions in which testosterone levels declined during culture under control (no BPA) conditions (Ben Maamar *et al.*, 2015). The demonstration that BPA effects are largely eliminated in the human fetal testis by addition of hLH in the media, indicates that under physiological conditions of pregnancy, in which gonadotrophin (hCG and/or LH) levels are extremely high, environmental exposure to BPA is unlikely to affect testosterone production in the human fetal testis (Eladak *et al.*, 2015). Moreover, a recent study has investigated the effects of alternative bisphenols that have been proposed as replacements for BPA (Eladak *et al.*, 2015). For unstimulated conditions, similar effects to those of BPA occurred following exposure to high concentrations of BPS, BPE and BPF, although the effects tended to be of lower magnitude and towards the higher concentrations compared to BPA (Eladak *et al.*, 2015; Gaudriault *et al.*, 2017).

AMH production from Sertoli cells appears to be unchanged as a result of BPA exposure in the human fetal testis (Ben Maamar *et al.*, 2015).

Insl3 is reduced following *in-vitro* exposure of human fetal testis to BPA (Ben Maamar *et al.*, 2015). This occurred only at the highest (10^−5^ M) and lowest (10^−8^ M) concentrations of BPA tested and also only under basal conditions. However, levels of Insl3 were generally much lower in these basal conditions than in media supplemented with hLH or hCG. For gonadotrophin supplemented cultures, BPA did not affect Insl3 production at any of the concentrations tested, similar to the results already described for testosterone (Ben Maamar *et al.*, 2015).

##### Germ cells

A recent study has investigated the effects of BPA exposure on germ cells in the human fetal testis. For first trimester testis (6–12 GW) using the *in-vitro* approach, exposure to 10^−5^ M BPA resulted in a significant increase in the number of apoptotic gonocytes after 72 h, although there was no effect on apoptosis at lower concentrations (Eladak *et al.*, 2018). In xenografts, long-term (5 weeks) exposure to high concentrations of BPA (10^−5^ M) in drinking water resulted in a modest (−19%) reduction in germ cells/mm^2^, indicating that a sustained increase in apoptosis of germ cells could reduce their number. Analysis of the individual germ cell populations demonstrated that there was a significant reduction in the proportion of germ cells expressing the gonocyte marker (AP2γ) with a reciprocal increase in pre-spermatogonial (MAGEA4) population. These findings are consistent with a reduction in gonocytes or alternatively an acceleration in the normal germ cell differentiation from gonocyte to pre-spermatogonium following BPA exposure (Eladak *et al.*, 2018).

#### Summary—bisphenols

Animal studies investigating effects of BPA exposure on fetal testis development have reported inconsistent results for indicators of testosterone production and germ cell development. Similarly, results of epidemiological studies investigating association between BPA and clinical indicators of reduced fetal testosterone (cryptorchidism and hypospadias) are inconsistent. For experimental studies involving human testis tissue, whilst the results of *in-vitro* experiments indicate the potential for BPA to reduce testosterone production from the fetal testis, this has been under basal conditions and xenograft studies have failed to demonstrate similar effects in either first or second trimester human fetal testis under gonadotrophin stimulation, as occurs in normal pregnancy. Furthermore, typical human exposure is likely to be well below the concentrations used for experimental studies involving animal(s) or human tissues based on meta-analysis and latest data on human BPA exposure using LC/MS/MS (Teeguarden *et al.*, 2016). Whilst germ cell effects have been described in one small study involving human fetal testis xenografts and relatively high BPA exposure (Eladak *et al.*, 2018), future studies should focus on exposures within the human-relevant range and on potential effects during different periods of gestation in order to determine the potential for effects on germ cells and future fertility in humans.

### Pesticides and fungicides

Pesticides and fungicides represent a heterogeneous group of chemicals widely used in agriculture. Many of these agents (e.g. Vinclozolin, Procymidone and prochloraz) are known to have anti-androgenic properties (Albert and Jegou, 2014). Whilst reproductive effects of many of these agents have been investigated in animal models (Taxvig *et al.*, 2013) and epidemiological studies (Bonde *et al.*, 2016), relatively few have been studied in experimental models involving the use of human tissues.

#### Animal studies

Several pesticides and fungicides have been proposed to be anti-androgenic with potential to impact testicular development and function in animal studies (Taxvig *et al.*, 2013). This includes *in-vivo* studies demonstrating effects of *in-utero* exposure to the fungicides Procymidone (Ostby *et al.*, 1999) or Prochloraz (Vinggaard *et al.*, 2005) on fetal testosterone production in experimental animals. Given that these agents are known to act as anti-androgens via interfering directly with the androgen receptor (Robitaille *et al.*, 2015), it can be hypothesized that effects occurring in rodent models might be more likely to be translated into human effects, as opposed to other agents (e.g. phthalates) in which the mechanism of action is less clear.

#### Epidemiology

Whilst a number of epidemiological studies have reported associations between *in-utero* exposure to selected pesticides and the development of male reproductive disorders (Bonde *et al.*, 2016), the majority of these studies involve different pesticides or exposure periods (e.g. outside the fetal period) to those investigated in experimental studies using human tissues. For studies in which human experimental data is available, epidemiological data exists only for Dieldrin (Damgaard *et al.*, 2006; Shen *et al.*, 2008). Higher concentrations of Dieldrin have been reported in the placentae of Danish compared to Finnish women. This correlates at the population level with the higher prevalence of cryptorchidism in Denmark than in Finland (Shen *et al.*, 2008); however, a study of Danish subjects from the same cohort did not identify a significant association between Dieldrin in breast milk and the development of cryptorchidism, when compared to controls (Damgaard *et al.*, 2006).

#### Experimental evidence from human studies

Experimental studies investigating the effects of exposure to pesticides on human fetal testicular development and function are limited. To date, only two studies have reported the effects of pesticide exposure on human fetal testis tissue/cells (Table IV). This includes a study investigating the effects of Dieldrin, an insecticide, on second trimester (14–16 GW) human fetal testis in an *in-vitro* system.

**Table IV:** Summary of experimental studies investigating effects of pesticide exposure in human fetal testis tissue.

Model and regimen							Results					
Exposure	Model	Fetal age (weeks)	Dose	Route	Regimen	Supplemented	Testosterone	AMH	INSL3	Germ cells	Study	Comments
4-Octylphenol	*In-vitro*	6–12	10^−5^ M	Media	3 weeks	Nil				↓	Bendsen *et al.* (2001)	
Atrazine	*In-vitro*	10–12	10^−9^ to 10^−5^ M	Media	72 h	hCG	↔				Gaudriault *et al.* (2017)	
Bitertanol	*In-vitro*	10–12	10^−7^ M	Media	72 h	hCG	↔				Gaudriault *et al.* (2017)	
	*In-vitro*	10–12	10^−6^ to 10^−5^ M	Media	72 h	hCG	↓ (50-75%)				Gaudriault *et al.* (2017)	
Chlordecone	*In-vitro*	10–12	10^−7^ to 10^−6^ M	Media	72 h	hCG	↔				Gaudriault *et al.* (2017)	
	*In-vitro*	10–12	10^−5^ to 10^−4^ M	Media	72 h	hCG	↓ (50–90%)				Gaudriault *et al.* (2017)	
Dieldrin	*In-vitro*	14–16	10^−12^ to 10^−9^ M	Media	24 h	Nil	↔	↔			Fowler *et al.* (2009)	
	*In-vitro*	14–16	10^−12^ to 10^−9^ M	Media	24 h	LH	↓ (30%)	↔			Fowler *et al.* (2009)	
Glyphosate	*In-vitro*	10–12	10^−8^ to 10^−4^ M	Media	72 h	hCG	↔				Gaudriault *et al.* (2017)	
Imazalil	*In-vitro*	10–12	10^−6^ M	Media	72 h	hCG	↔				Gaudriault *et al.* (2017)	
	*In-vitro*	10–12	10^−5^ to 10^−4^ M	Media	72 h	hCG	↓ (50–100%)				Gaudriault *et al.* (2017)	
Ortho-PhenylPhenol	*In-vitro*	10–12	10^−7^ to 10^−5^ M	Media	72 h	hCG	↔				Gaudriault *et al.* (2017)	
	*In-vitro*	10–12	10^−4^ M	Media	72 h	hCG	↓ (40%)				Gaudriault *et al.* (2017)	
Prochloraz	*In-vitro*	10–12	10^−8^ M	Media	72 h	hCG	↔				Gaudriault *et al.* (2017)	
	*In-vitro*	10–12	10^−7^ to 10^−6^ M	Media	72 h	hCG	↓ (20–75%)				Gaudriault *et al.* (2017)	
Propiconazole	*In-vitro*	10–12	10^−7^ M	Media	72 h	hCG	↔				Gaudriault *et al.* (2017)	
	*In-vitro*	10–12	10^−6^ to 10^−5^ M	Media	72 h	hCG	↓ (40–75%)				Gaudriault *et al.* (2017)	

Significant effects associated with adverse outcomes are highlighted in red, no change or significant effects not expected to result in adverse outcomes are highlighted in green.

##### Hormones

Exposure to concentrations of Dieldrin relevant to environmental and maternal serum levels in pregnancy (10^−12^ M to 10^−9^ M) did not affect testosterone production under basal conditions; however, testosterone (−30%) and gene expression of the steroidogenic enzyme ‘steroidogenic acute regulatory protein’ (StAR) were significantly reduced under LH-induced conditions (Fowler *et al.*, 2007). No effect of Dieldrin exposure was found on AMH production from Sertoli cells. A more recent study investigated the effect of a wide variety of chemicals, including pesticides, on testosterone production in human fetal testis using an *in-vitro* culture system (Gaudriault *et al.*, 2017). This study identified dose-dependent reductions in testosterone as a result of exposure to a range of pesticides including imazalil, propiconazole, bitertanol, prochloraz, chlordecone, and for the majority of these chemicals, exposure to ~10^−5^ M resulted in a 50% reduction in testosterone production compared to basal. For glyphosate and atrazine, there was no clear reduction in testosterone across the entire dose range and for ortho-phenylphenol, a reduction in testosterone was only demonstrated at the highest concentration (10^−4^ M).

##### Germ cells

Potential effects of exposure to pesticides and antifungals on germ cell development in the human fetal testis have been investigated in one study (Bendsen *et al.*, 2001). The effects of 4-octylphenol, a surfactant used as a component of several pesticides, were investigated in prolonged (3 weeks) culture of first trimester (6–12 GW) human fetal testis tissue. Exposure resulted in a significant decrease in the mitotic index and in the number of spermatogonia per unit area compared to vehicle-exposed controls (Bendsen *et al.*, 2001).

#### Summary—pesticides and fungicides

Evidence for effects of several pesticides have been described in a limited number of experimental studies using human fetal testis tissues. The majority of the agents investigated thus far have demonstrated a reduction in testosterone production following short term *in-vitro* culture. However, the relationship between the concentrations used for each individual agent and measures of human exposure remain to be elucidated. Furthermore, the possibility of correlating the results of epidemiological and experimental studies is hindered by the variation in agents investigated by each approach.

## Pharmaceuticals

Over recent years, there has been increasing interest in the potential effects of pharmaceutical exposure during pregnancy on reproductive development and subsequent reproductive function in the offspring. The use of pharmaceuticals during pregnancy results in direct exposure to mother and the developing fetus. Therefore exposure to relatively high concentrations of the circulating drug may result, which contrasts with the very low level exposure to environmental chemicals such as those described above. In addition, for some pharmaceuticals (e.g. analgesics) the frequency of use during pregnancy may be high resulting in sustained exposures.

### Analgesics

Analgesics are the most commonly used medications worldwide. Several of these are available without prescription and it has been reported in many countries that 50–90% of women will use an analgesic at some stage during pregnancy (Kristensen *et al.*, 2016). Paracetamol (acetaminophen) is the most commonly used analgesic, whilst non-steroidal anti-inflammatory drugs (NSAIDs) including ibuprofen are also used by up to 15% of pregnant women (Kristensen *et al.*, 2016).

#### Animal studies

Paracetamol exposure reduced fetal androgen production in rodents in studies using both *in-vivo* and *in-vitro* approaches (Kristensen *et al.*, 2011, 2012; Axelstad *et al.*, 2014; Holm *et al.*, 2015; van den Driesche *et al.*, 2015a), similar to findings for aspirin exposure following *in-vivo* (20% reduction in AGD) (Gupta and Goldman, 1986) or *in-vitro* (70% reduction in testosterone) (Kristensen *et al.*, 2012) exposure. For indomethacin exposure in mice, results are conflicting in terms of fetal testosterone production, with one *in-vivo* study describing a 20% reduction in AGD (Gupta and Goldman, 1986), whilst rat studies described either no effect *in vivo* (Dean, Mungall *et al.*, 2013), or a 30% reduction in fetal testosterone production *in vitro* (Kristensen *et al.*, 2012).

#### Epidemiology

The majority of studies that investigated associations between maternal analgesic exposure and cryptorchidism in male offspring have reported a significant positive association (Berkowitz and Lapinski, 1996; Jensen *et al.*, 2010; Kristensen *et al.*, 2011; Snijder *et al.*, 2012), although this is not a consistent finding (Philippat *et al.*, 2011). The timing of maternal exposure may be a key factor as most associations are reported to occur following prolonged exposure (>4 weeks) (Jensen *et al.*, 2010) or during the second trimester (Hurtado-Gonzalez and Mitchell, 2017), which would coincide with at least part of the postulated critical human MPW period (8–14 GW) (Welsh *et al.*, 2008). A reduction in fetal testosterone production, as demonstrated in experimental studies described above, could provide a mechanistic explanation for paracetamol-induced cryptorchidism in male offspring, although proving this in humans is challenging. However, measurement of the AGD in offspring can provide an indirect read-out of fetal androgen production, linking the reported association with the proposed mechanism (Dean and Sharpe, 2013). A recent study has shown an association between paracetamol exposure (inclusive of the MPW) and reduced AGD in boys up to 24 months, independent of body size (Fisher *et al.*, 2016). Nevertheless, for these epidemiological studies, extrapolation of results to direct clinical effects should be considered with caution, primarily due to the lack of direct analgesic measurements and the reliance on retrospective questionnaires for exposure classification which may involve a degree of recall bias.

#### Experimental evidence from human studies

Several recent studies have investigated the effect of analgesics on human fetal testis using experimental models (Table V).

**Table V:** Summary of experimental studies investigating effects of analgesic exposure in human fetal testis tissue.

Model and regimen							Results					
Exposure	Model	Fetal age (weeks)	Dose	Route	Regimen	Supplemented	Testosterone	AMH	INSL3	Germ Cells	Study	Comments
Paracetamol	*In-vitro*	8–11	10^−5^ M	Media	7d	Nil				↓	Hurtado-Gonzalez *et al.* (2018)	
	*In-vitro*	8–12	10^−5^ M	Media	24–72 h	hCG	↔		↓*	↔	Mazaud-Guittot *et al.* (2013)	*Dose response ↓
	*In-vitro*	10–12	10^−5^ to 10^−4^ M	Media	72 h	hCG	↔				Gaudriault *et al.* (2017)	
	*In-vitro*	10–12	10^−8^ to 10^−6^ M	Media	72 h	hCG	↑ (25%)				Gaudriault *et al.* (2017)	
	Xenograft	14–20	60 mg/kg/d	Oral Gavage	7d	hCG	↓				van den Driesche *et al.* (2015a)	
	Xenograft	14–20	60 mg/kg/d	Oral Gavage	7d	hCG				↓	Hurtado-Gonzalez *et al.* (2018)	
	Xenograft	14–20	60 mg/kg/d	Oral Gavage	1d	hCG	↔				van den Driesche *et al.* (2015a)	
	Xenograft	14–20	60 mg/kg/d	Oral Gavage	1d	hCG				↓	Hurtado-Gonzalez *et al.* (2018)	
Ibuprofen	Xenograft	14–17	60 mg/kg/d	Oral Gavage	7d	hCG	↔	↔			Ben Mamaar (2017)	
	Xenograft	14–20	60 mg/kg/d	Oral Gavage	1d	hCG				↓	Hurtado-Gonzalez *et al.* (2018)	
	Xenograft	14–20	60 mg/kg/d	Oral Gavage	7d	hCG				↓	Hurtado-Gonzalez *et al.* (2018)	
	*In-vitro*	7–8	10^−5^ M	Media	72 h	hCG	↔	↓			Ben Mamaar (2017)	
	*In-vitro*	8–10	10^−5^ to 10^−4^ M	Media	72 h	hCG	↓	↓	↔	↔	Ben Mamaar (2017)	
	*In-vitro*	8–10	10^−7^ to 10^−6^ M	Media	72 h	hCG	↔	↓^**^	↔		Ben Mamaar (2017)	**10^−7^ only
	*In-vitro*	8–11	10^−5^ M	Media	7d	Nil				↓***	Hurtado-Gonzalez *et al.* (2018)	***Gonocytes only
	*In-vitro*	10–12	10^−7^ to 10^−4^ M	Media	24–72 h	hCG	↔	↔*	↔*		Ben Mamaar (2017)	*Dose response ↓
	*In-vitro*	10–12	10^−8^ to 10^−5^ M	Media	72 h	hCG	↔				Gaudriault *et al.* (2017)	
	*In-vitro*	10–12	10^−4^ M	Media	72 h	hCG	↑				Gaudriault *et al.* (2017)	
Aspirin	*In-vitro*	8–10	10^−7^ M	Media	72 h	hCG	↔				Mazaud-Guittot *et al.* (2013)	
	*In-vitro*	8–10	10^−6^ to 10^−4^ M	Media	72 h	hCG	↑	↑^#^		↔	Mazaud-Guittot *et al.* (2013)	^#^Only tested at 10^−5^ (8–12 GW)
	*In-vitro*	10–12	10^−7^ to 10^−4^ M	Media	72 h	hCG	↔		↔^##^		Mazaud-Guittot *et al.* (2013)	^##^Only tested at 10^−5^
	*In-vitro*	10–12	10^−8^ to 10^−4^ M	Media	72 h	hCG	↔				Gaudriault *et al.* (2017)	
Indomethacin	*In-vitro*	8–12	10^−5^ M	Media	72 h	hCG	↑ (20%)		↔^###^	↔	Mazaud-Guittot *et al.* (2013)	^###^(10–12 GW)
	*In-vitro*	10–12	10^−8^ to 10^−5^ M	Media	72 h	hCG	↔				Gaudriault *et al.* (2017)	
	*In-vitro*	10–12	10^−4^ M	Media	72 h	hCG	↑ (20%)				Gaudriault *et al.* (2017)	
Aniline	*In-vitro*	10–12	10^−8^ to 10^−5^ M	Media	96 h	hCG	↔^####^				Gaudriault *et al.* (2017)	^####^↓ 20% (10^−7^)

Significant effects associated with adverse outcomes are highlighted in red, no change or significant effects not expected to result in adverse outcomes are highlighted in green.

##### Paracetamol - Hormones

*In-vitro* studies using first trimester testis (8–12 GW) exposed to paracetamol (10^−5^ M) for 1–3 days did not alter testosterone production, compared with vehicle controls (Mazaud-Guittot *et al.*, 2013). This was also the case for the paracetamol metabolite AM404 (10^−5^ M) (Mazaud-Guittot *et al.*, 2013). A recent study using an organotypic *in-vitro* culture system of first trimester human fetal testes explants showed that exposure to paracetamol in a dose range of 10^−8^ to 10^−6^ M increased testosterone by 25%; however, higher doses of 10^−5^ M and 10^−4^ M did not have any effect on testosterone production (Gaudriault *et al.*, 2017).

The conflicting results between rodent and human *in-vitro* studies may relate to the stage of testis development (i.e. timing within the MPW) or differences in the experimental system (Mazaud-Guittot *et al.*, 2013). Caution should be exercised when relating effects using *in-vitro* models to the *in-vivo* situation in humans, as the former cannot directly recapitulate normal pharmacokinetics, including *in-vivo* peak and trough concentrations. To circumvent some of the potential limitations of the *in-vitro* approaches for the human fetal testis, subsequent studies have utilized an *ex-vivo* approach involving subcutaneous xenografting of human fetal testis tissues (*n* = 5; 14–20 GW) into host castrate nude mice. Oral exposure of these mice to a human-relevant regimen of paracetamol (20 mg/kg; three times daily) for 1 week significantly reduced (−45%) host serum testosterone and seminal vesicle (androgen dependent organ) weight (−18%), unlike a single daily exposure which had no effect on either parameter (van den Driesche *et al.*, 2015a). Further confirmation of the human relevance of paracetamol exposure is evident from the finding that 1 h after the final dose in host mice, plasma paracetamol concentrations were significantly lower than post-therapeutic levels reported in pregnant women (Rayburn *et al.*, 1986). However, it must be considered that circulating paracetamol levels in pregnant women may not be a direct indicator of intra-testicular levels in the developing fetus.

Insl3 hormone production was significantly reduced in first trimester human fetal testis cultures exposed to paracetamol, in which a clear dose–response relationship with increasing paracetamol exposure (at 10^−7^ M to 10^−4^ M) was demonstrated (Mazaud-Guittot *et al.*, 2013).

##### Paracetamol - Germ cells

An *in-vitro* study using first trimester human fetal testis (8–12 GW) exposed to paracetamol (10^−5^ M) for 1–3 days found no alteration in germ cell number (Mazaud-Guittot *et al.*, 2013). However, a more recent *in vitro* study with a longer period (7 days) of exposure showed that similar paracetamol concentration (10^−5^ M) significantly reduced (−28%) gonocyte number (Hurtado-Gonzalez *et al.*, 2018). The differing findings in these studies may reflect the longer period of exposure in the latter study or may be the result of differences in the culture systems. To further investigate the potential effect of paracetamol exposure on the human fetal testis, a xenograft approach was also used alongside the *in-vitro* model (Hurtado-Gonzalez *et al.*, 2018). Xenografted second trimester tissue (14–20 GW) exposed to paracetamol using a human-relevant exposure regimen (20 mg/kg, three times daily) resulted in a reduction in gonocyte number after 7 days exposure (−32%). Interestingly, a reduction in gonocyte number (−17%) was also demonstrated after just 1 day of exposure to paracetamol (Hurtado-Gonzalez *et al.*, 2018).

Whilst most of circulating paracetamol in humans comes from use of paracetamol-containing medications, an alternative source has also been described. The industrial chemical aniline, which is found in a wide variety of manufactured products, such as rubber, pharmaceuticals, cosmetics and cigarette smoke, is rapidly metabolized to paracetamol inside the body (Modick *et al.*, 2014, 2016). Furthermore, *in-vivo* studies in which male mice exposed *in utero* to aniline have shown similar fetal anti-androgenic effects to those described for exposure to paracetamol (Holm *et al.*, 2015). Only one study has investigated the effect of aniline on the human fetal testis (Gaudriault *et al.*, 2017). *In-vitro* exposure of first trimester (10–12 GW) human fetal testis to aniline for 96 h had no effect on testosterone production across a range of doses (10^−8^ M to 10^−5^ M) apart from a small reduction (−20%) for an intermediate concentration (10^−7^ M) (Gaudriault *et al.*, 2017).

##### Ibuprofen - Hormones

Exposure of first trimester human fetal testis explants to ibuprofen using an organotypic culture system did not affect testosterone production (Gaudriault *et al.*, 2017). However, another *in-vitro* study reported a reduction in steroidogenic enzyme expression across a similar range of concentrations (10^−4^ M, 10^−5^ M). This effect was only evident for early first trimester testes (8–9 GW), as there was no effect at any other gestational time-point examined (<8 GW or >10 GW) (Ben Maamar *et al.*, 2017). Similarly, there was no effect of exposure to ibuprofen on testosterone production in host mice carrying xenografts of second trimester human fetal testis tissue (Ben Maamar *et al.*, 2017).

Ibuprofen exposure for 3 days in an *in-vitro* model reduced AMH in first trimester human fetal testes at 7–8 GW (10^−5^ M), and at 8–10 GW (10^−4^ M to 10^−5^ M); however, no significant difference in AMH was found after 7 days of exposure of host mice carrying second trimester human fetal testis xenografts (Ben Maamar *et al.*, 2017).

Insl3 production was not affected in early first trimester (8–10 GW) human fetal testis following *in-vitro* culture with ibuprofen, whilst for late first trimester (10–12 GW) testis, an overall dose response reduction was demonstrated (Ben Maamar *et al.*, 2017). The fact that ibuprofen exposure affects testosterone and Insl3 production only during specific periods of human fetal testis development, has implications for the potential of this analgesic to impact testis descent, i.e. cryptorchidism (which is under the control of these two hormones).

##### Ibuprofen - Germ cells

*In-vitro* culture and exposure of first trimester human fetal testis to ibuprofen for 7 days resulted in a reduction in gonocyte number (−22%). However, there were no significant changes to germ cell number following exposure of second trimester xenografted testis tissue (Hurtado-Gonzalez *et al.*, 2018).

##### Aspirin - Hormones

Aspirin exposure of first trimester testis explants in an organotypic culture system, did not alter testosterone production across a range of concentrations (10^−4^ to 10^−8^ M) (Gaudriault *et al.*, 2017). However, in a separate *in-vitro* culture study, a significant dose–response relationship was reported whereby aspirin exposure for 3 days significantly increased the production of testosterone by early first trimester (8–9 GW), but not in late first trimester (10–12 GW) testes (Mazaud-Guittot *et al.*, 2013). *In-vitro* exposure of human fetal testis (10–12 GW) to aspirin for 3 days did not affect Insl3 production (Mazaud-Guittot *et al.*, 2013), whereas AMH production was significantly increased.

##### Aspirin - Germ cells

*In-vitro* exposure to aspirin did not affect germ cell number in first trimester (8–10 GW) human fetal testis tissue (Mazaud-Guittot *et al.*, 2013).

##### Indomethacin - Hormones

Similar to rodent studies, there are conflicting results of experimental studies using *in-vitro* culture of human fetal testis. Exposure of first trimester human fetal testis explants (10–12 GW) to indomethacin (10^−4^ M) for 72 h, reduced testosterone (−20%), whereas exposure at lower concentrations (10^−5^ M to 10^−8^ M) had no effect (Gaudriault *et al.*, 2017). In contrast, a previous study found that indomethacin exposure (10^−5^ M) increased testosterone production (+20%), when first trimester (8–12 GW) testes were exposed *in-vitro* for a similar duration (Mazaud-Guittot *et al.*, 2013). *In-vitro* exposure of human fetal testis (10–12 GW) to indomethacin (10^−5^ M) for 2 days did not affect Insl3 production (Mazaud-Guittot *et al.*, 2013).

##### Indomethacin - Germ cells

*In-vitro* exposure to indomethacin (10^−5^ M) for 2 days did not affect germ cell number in first trimester (8–12 GW) human fetal testis cultures (Mazaud-Guittot *et al.*, 2013).

#### Summary—analgesics

Exposure to analgesics has been linked to abnormalities in testicular function and development of male reproductive disorders across a range of studies. This includes epidemiological and experimental studies using animal and human tissues. Results regarding testosterone production are not consistent and this may reflect differences between species, model systems or dose, timing and duration of exposure (Table I and Fig. 2). However, there is increasing evidence from human studies that paracetamol and ibuprofen can affect germ cell number in the fetal testis and evidence exists for similar effects on germ cells in the fetal ovary (Hurtado-Gonzalez *et al.*, 2018). These studies involve exposure to human-relevant concentrations of these drugs and for xenograft studies they include comparable dosing regimens to those used therapeutically in humans. Whilst the evidence for effects of paracetamol and ibuprofen on germ cells appears robust, the potential for such exposures to impact subsequent male reproductive function and fertility are unexplored. Indeed, it is possible that there may be compensation later in gestation or during childhood that would rescue the effects of any fetal germ cell loss. Large-scale prospective epidemiological studies and longer-term experimental (e.g. xenograft) studies can help to address this particular question.

### Diethylstilboestrol

Diethylstilboestrol (DES) is a synthetic estrogen that was used clinically to prevent spontaneous miscarriage and pre-term labor from the 1940 s until the early 1970 s (Marselos and Tomatis, 1992). DES was withdrawn from clinical use after the demonstration of a causal role in the development of vaginal carcinoma in girls born to exposed mothers (Herbst *et al.*, 1971). In addition to the effects on female offspring, an association with structural abnormalities of the male reproductive tract was also described including epididymal cysts, microphallus and testicular hypoplasia (Toppari *et al.*, 1996).

#### Animal studies

Animal studies involving *in-vitro* culture of rat and mouse fetal testis, have reported a reduction in testosterone production following exposure to DES (N’Tumba-Byn *et al.*, 2012), similar to the results of previous *in-vitro* studies involving fetal mice (Delbes *et al.*, 2005) and *in-vivo* studies in rats (Haavisto *et al.*, 2001).

#### Epidemiology

For TDS disorders, which are linked to a reduction in androgen action during fetal life, there is conflicting evidence regarding their association with maternal DES exposure. Three studies have reviewed the literature relating to exogenous estrogen exposure and male reproductive disorders (Toppari *et al.*, 1996; Storgaard *et al.*, 2006; Martin *et al.*, 2008). Whilst early studies reported that hypospadias was significantly associated with DES exposure (Henderson *et al.*, 1976), it has subsequently been pointed out that this related to urethral abnormalities resulting from exposure to exogenous estrogens (including DES), which may have resulted from abnormalities in penile development rather than an effect on urethral formation as a result of reduced androgen exposure (Joffe, 2002). The meta-analysis of all available evidence revealed a significant association between DES exposure and hypospadias; however, it was concluded that any effect of DES on hypospadias is likely to be small (Martin *et al.*, 2008). For cryptorchidism, an increased risk in association with DES exposure is reported; however, this was dependent on the statistical model used and was indicative of heterogeneity (Martin *et al.*, 2008). A subsequent cohort study has reported an association between *in-utero* exposure to DES and an increased risk of cryptorchidism, however, only for those in whom the initial exposure occurred prior to the 11th week of gestation with no significant association following exposure after 11 GWs (Palmer *et al.*, 2009). Studies have demonstrated no effect of prenatal DES exposure on sperm counts (Leary *et al.*, 1984) or fertility (Wilcox *et al.*, 1995); however, this is in contrast to a previous study demonstrating an association between prenatal exposure to DES and semen parameters in adult men (Gill *et al.*, 1979). Importantly, this study included analysis of men born to a large cohort of mothers who participated in an RCT involving DES exposure during pregnancy.

#### Experimental evidence from human studies

To date, only two studies have investigated the effect of DES exposure on the human fetal testis (Table VI). *In-vitro* organ culture of first trimester human fetal testis exposed to DES (10^−5^ to 10^−6^ M) for 3 days did not alter testosterone production (N’Tumba-Byn *et al.*, 2012). Interestingly, this study compared effects of DES exposure in rodent and human fetal testis demonstrating contrasting results between species using an identical experimental system (N’Tumba-Byn *et al.*, 2012).

In a separate study using the xenograft model, exposure to DES (100μg/kg, three times weekly) for 35 days resulted in no significant difference in testosterone production by second trimester (15–19 GW) testis tissue. Interestingly, host mouse seminal vesicles were significantly increased in weight, which was indicative of increased testosterone production from the xenografted tissue over the entire grafting period (Mitchell *et al.*, 2013). The reason for this unexpected increase in testosterone is unclear.

#### Summary—diethylstilboestrol

Whilst rodent studies have indicated a profoundly negative effect of DES exposure on testosterone production by the fetal testis (Haavisto *et al.*, 2001; Delbes *et al.*, 2005; N’Tumba-Byn *et al.*, 2012), experimental studies utilizing human fetal testis tissues have failed to identify similar effects (N’Tumba-Byn *et al.*, 2012; Mitchell *et al.*, 2013), which may relate to the presence of ESR1 in rodent Leydig cells, and the absence of this estrogen receptor in human fetal testis (Mitchell *et al.*, 2013). Epidemiological data suggests that any potential effect of DES exposure on male reproductive development is likely to be of small magnitude. Taken together the results suggest an important species difference in terms of DES effects on fetal testosterone production which may explain why this frequently results in the development of male reproductive disorders in rodents, whilst associations between DES and subsequent male reproductive disorders in humans are rather modest. Whilst DES is unlikely to be used in pregnant women in the future, the findings of this study offer some reassurance regarding the potential of low-level exposure to environmental estrogens to affect human male reproductive development, given their extremely low potency compared with DES and the high exposures that resulted from therapeutic use of DES.

### Metformin

Metformin is a biguanide used as an insulin sensitizer in the treatment of Type 2 Diabetes and obesity. Metformin may also be prescribed during pregnancy in individuals with pre-existing diabetes or those that have developed gestational diabetes during their pregnancy (https://bnf.nice.org.uk/drug/metformin-hydrochloride.html#pregnancy).

#### Animal studies

Exposure to metformin has been shown to reduce testosterone production in mouse fetal testis following *in-vitro* or *in-vivo* exposure. Exposure of pregnant mice to metformin (300 mg/gk/d) from embryonic day (e) 0.5 resulted in a significant reduction in testosterone at e16.5 (Tartarin *et al.*, 2012).

#### Epidemiology

To date, there has been no epidemiological data relating *in-utero* exposure to metformin to TDS disorders at birth, although one study found no association between prepubertal testicular volumes in offspring born to mothers who had received metformin, compared with insulin, for gestational diabetes (Tertti *et al.*, 2016).

#### Experimental evidence from human studies

A recent study has investigated the effect of metformin exposure on the human fetal testis using an *in-vitro* culture system (Tartarin *et al.*, 2012) (Table VI). Exposure to a range of metformin concentrations (5×10^−5^M to 5×10^−3^M) resulted in a significant decrease in testosterone production from the testis. Importantly, the lowest concentration (5×10^−5^M) reflects the serum levels measured in humans following a therapeutic dose of metformin (Robert *et al.*, 2003).

**Table VI:** Summary of experimental studies investigating effects of pharmaceutical exposure in human fetal testis tissue.

Model and regimen							Results					
Exposure	Model	Fetal age (weeks)	Dose	Route	Regimen	Supplemented	Testosterone	AMH	INSL3	Germ cells	Study	Comments
DES	*In-vitro*	6–12	10^−6^ to 10^−5^ M	Media	72 h	Nil	↔				N-Tumba Byn (2012)	
	Xenograft	15–19	100 ug	Subcut.	3×/wk for 35 d	hCG	↔*				Mitchell *et al.* (2013)	*↑ SV (60%)
Metformin	*In-vitro*	10–12	5 × (10^−5^ to 10^−3^) M	Media	72 h	n/a	↓ (30–70%)**				Tartarin *et al.* (2012)	**5 × 10^−5^ and 5 × 10^−3^ M
Abiraterone	Xenograft	16–22	75mg/kg/day	Oral	14 d	hCG	↓ (80%)**			↔	Spade *et al.* (2014)	***↓ SV (45%)
Ketoconazole	*In-vitro*	10–12	10^−7^ M	Media	96 h	hCG	↔				Gaudriault *et al.* (2017)	
	*In-vitro*	10–12	10^−6^ to 10^−5^ M	Media	96 h	hCG	↓ (50–90%)				Gaudriault *et al.* (2017)	
	*In-vitro*	8–12	10^−5^ M	Media	24–72 h	hCG	↓ (20–90%)	↓ (65%)^#^	↓ (90%)		Mazaud-Guittot *et al.* (2013)	^#^72 h
Theophylline	*In-vitro*	10–12	10^−7^ to 10^−5^ M	Media	96 h	hCG	↓ (30–40%)				Gaudriault *et al.* (2017)	
Valproate	*In-vitro*	10–12	10^−7^ to 10^−6^ M	Media	96 h	hCG	↔				Gaudriault *et al.* (2017)	
	*In-vitro*	10–12	10^−5^ M	Media	96 h	hCG	↓ (65%)				Gaudriault *et al.* (2017)	
Clomiphene	*In-vitro*	10–12	10^−6^ to 10^−5^ M	Media	96 h	hCG	↓ (25–65%)				Gaudriault *et al.* (2017)	

Significant effects associated with adverse outcomes are highlighted in red, no change or significant effects not expected to result in adverse outcomes are highlighted in green.

### Azole antifungals and abiraterone

Azole antifungals (e.g. ketoconazole, fluconazole) and abiraterone are drugs known to inhibit key enzymes of the steroidogenic pathway including P450scc (CYP17A1; ketoconazole) and CYP17A1 & 3βHSD (abiraterone). As a result, exposure of the fetal testis to these agents could be predicted to affect testosterone production and potentially to result in the development of male reproductive disorders.

#### Epidemiology

Exposure to ‘azole’ antifungals that interfere with steroidogenesis may be relevant to pregnancy, given that they are frequently prescribed for the treatment of vaginal candidiasis. No association between maternal use of antifungals and hypospadias has been reported in two studies (Carter *et al.*, 2008) (Norgaard *et al.*, 2008), or for AGD after exposure to antifungals administered as vaginal tablets or as topical cream (Mogensen *et al.*, 2017). However, the latter study demonstrated a significant association between oral fluconazole and reduced AGD in the male offspring (Mogensen *et al.*, 2017). Importantly, for each of these three studies, the numbers of cases was small and larger prospective studies would be required to provide definitive evidence of associations between antifungals and indicators of fetal testosterone production.

#### Experimental evidence from human studies

Exposure to ketoconazole results in a significant reduction (50–90%) in testosterone following *in-vitro* exposure of first trimester human fetal testis for 96 h (Gaudriault *et al.*, 2017) (Table VI). Similar results have been described for ketoconazole with a progressive reduction in testosterone production after 24 h (−20%), 48 h (−90%) and 72 h (−95%), compared to vehicle-exposed tissue (Mazaud-Guittot *et al.*, 2013). This study also reported a ketoconazole-induced reduction in INSL3 (−100%) and AMH (−50%) after 72 h of culture.

Abiraterone (an anti-androgen used in prostate cancer) has also been shown to result in a reduction in testosterone production (−80%) in second trimester human fetal testis xenografts (grafted for 14 days), whilst no effect on germ cell number was demonstrated (Spade *et al.*, 2014) (Table VI). Indeed, these agents may be considered as positive controls for studies investigating the effects of exposures on testosterone production in human fetal testes (Mazaud-Guittot *et al.*, 2013; Spade *et al.*, 2014).

### Other pharmaceuticals

A recent study has described the effect of exposure to 27 different chemicals, including several additional pharmaceuticals, on the human fetal testis using an *in-vitro* system (Gaudriault *et al.*, 2017) (Table VI). A dose dependent reduction in testosterone production was determined for clomiphene (an anti-estrogenic substance used to stimulate ovulation), theophylline (a methylxanthine drug which acts as a non-selective phosphodiesterase inhibitor used in asthma) and valproate (an anti-epileptic) following *in-vitro* culture of human fetal testis tissue for 96 h (Gaudriault *et al.*, 2017). Interestingly, valproate has been associated with hypospadias in male offspring of exposed mothers (Veroniki *et al.*, 2017).

## Lifestyle

Exposures that relate to lifestyle may also impact on the development of male reproductive disorders. However, to date only a limited number of studies using human fetal tissues have been conducted in this area (Table VII).

**Table VII:** Summary of experimental studies investigating effects of lifestyle exposures in human fetal testis tissue.

Model and regimen							Results					
Exposure	Model	Fetal age (weeks)	Dose	Route	Regimen	Supplemented	Testosterone	AMH	INSL3	Germ cells	Study	Comments
1,3,7 TMUA	*In-vitro*	10–12	10^−9^ to 10^−5^ M	Media	72 h	hCG	↔*				Gaudriault *et al.* (2017)	*↓ at 10^−7^ M
Alcohol	*In-vitro*	10–12	10^−8^ to 10^−5^ M	Media	72 h	hCG	↑ (225–275%)				Gaudriault *et al.* (2017)	Ethanol
Caffeine	*In-vitro*	10–12	10^−9^ M	Media	72 h	hCG	↓ (20%)				Gaudriault *et al.* (2017)	
	*In-vitro*	10–12	10^−7^ to 10^−5^ M	Media	72 h	hCG	↔				Gaudriault *et al.* (2017)	
Paraxanthine	*In-vitro*	10–12	10^−9^ to 10^−5^ M	Media	72 h	hCG	↔				Gaudriault *et al.* (2017)	
Smoking	*In-vitro*	14–19	10^−6^ M	Media	24 h	Nil				↑ Apoptosis	Coutts *et al.* (2007)	DMBA-DHD
	*In-vitro*	7-11	10^−7^ to 10^−5^ M	Media	72 h	Nil	↔			↓ (20%)**	Angenard *et al.* (2010)	Cadmium; **10^−6^ M only
Theobromine	*In-vitro*	10–12	10^−9^ to 10^−5^ M	Media	72 h	hCG	↔***				Gaudriault *et al.* (2017)	***↓ at 10^−7^ M

Significant effects associated with adverse outcomes are highlighted in red, no change or significant effects not expected to result in adverse outcomes are highlighted in green.

### Xanthines

Xanthines are a class of compounds that share a common structure and have stimulant properties, including caffeine, one of the most commonly used recreational drugs worldwide. Effects of several of these compounds have been investigated *in vitro* (Gaudriault *et al.*, 2017).

#### Experimental evidence from human studies

No negative effects on testosterone production from cultured human fetal testis tissue were demonstrated following exposure to caffeine, paraxanthine, theobromine or 1,3,7 trimethyluric acid (TMUA), albeit there appeared to be a modest decrease in testosterone production for caffeine only at the lowest concentration (Table VII).

### Alcohol

Alcohol exposure during pregnancy is known to impact on fetal development, and its mechanism of action could occur centrally or in the placenta. Fetal alcohol syndrome is well described and can include neurodevelopmental disorders, facial dysmorphism and growth abnormality (Nash and Davies, 2017).

#### Epidemiology

An epidemiological study involving a Danish–Finnish cohort (~2500 boys), demonstrated an association between maternal alcohol consumption and increased risk of cryptorchidism in sons (Damgaard *et al.*, 2007).

#### Experimental evidence from human studies

The effect of exposure to alcohol on human fetal testis has only been investigated in one study (Table VII). Interestingly, exposure of first trimester human fetal testis to ethanol (10^−8^ M to 10^−5^ M for 72 h) using *in-vitro* culture resulted in a significant increase in testosterone production across a wide dose range (Gaudriault *et al.*, 2017).

### Smoking

Maternal smoking is known to have many potentially harmful effects on the developing fetus including intra-uterine growth retardation and low birth weight (Abraham *et al.*, 2017).

#### Epidemiology

Epidemiological studies have measured sperm counts in men exposed *in-utero* to maternal cigarette smoke, which demonstrated a reduction in sperm concentration (38–48%) in exposed- compared to unexposed-men (Storgaard *et al.*, 2003; Ramlau-Hansen *et al.*, 2007). Use of nicotine substitutes during pregnancy significantly increased the risk of cryptorchidism in male offspring (Damgaard *et al.*, 2008) and an association between maternal smoking during pregnancy and cryptorchidism in male offspring has also been demonstrated (Jensen *et al.*, 2007).

#### Experimental evidence from human studies

##### Hormones

One study compared testosterone levels in plasma of human fetuses exposed to maternal cigarette smoke with those of non-smoking mothers and found no difference in testosterone between the groups despite a significant reduction in hCG (Fowler *et al.*, 2009).

##### Germ cells

The effects of maternal smoking on human fetal testis has been investigated in two experimental studies, both of which used exposure to components of cigarette smoke (Coutts *et al.*, 2007; Angenard *et al.*, 2010). *In-vitro* exposure of human fetal testis to DMBA-DHD, the active metabolite of polyaromatic hydrocarbons found in cigarette smoke, resulted in a significant increase in apoptosis in germ cells, which could be rescued by antagonism of the aryl hydrocarbon receptor (AHR), indicating that activation of the AHR is the likely mechanism for the effects of DMBA-DHD on germ cells (Coutts *et al.*, 2007). Cadmium (another component of cigarette smoke)-exposure of first trimester human fetal testis resulted in increased apoptosis in germ cells, without any effect on testosterone production (Angenard *et al.*, 2010).

### Mixtures

Over recent years, it has been increasingly recognized that the impact of environmental exposures depends not only on the individual agents, but also on the combination of agents. A number of animal studies have investigated the effects of ‘mixtures’; however, to date such approaches using human fetal testis tissues is limited. A key aspect of this is whether the effects can be considered as additive or synergistic. The effect of four separate mixtures (all including BPA) has been investigated in a recent study involving *in-vitro* exposure of first trimester human fetal testis for 96 h (Gaudriault *et al.*, 2017). This included two mixtures (four agents each) of BPA + pharmaceuticals and two mixtures (eight agents each) which included additional environmental (pesticides and bisphenols) chemicals.

As expected, each mixture resulted in a reduction in testosterone production. Importantly, the authors compared the individual dose–response results to those predicted by the additive effect of the individual agents. There was a high correlation between predicted and actual response for each of the four mixtures indicating that these agents acted in an additive manner (Gaudriault *et al.*, 2017). This allows such experimental systems to use the results of exposure to individual agents for approximation of the combined anti-androgenic effect of multiple exposures, based on an assumption of dose-addition. This has important implications for informing regulation of environmental chemicals and pharmaceutical exposures.

## Discussion

Whilst a relatively large body of animal data exists for determining the impacts of *in-utero* exposures on fetal testicular development and male reproductive disorders, a limited number of experimental studies involving human tissues have been conducted. Animal models offer the potential to conduct more expansive studies involving exposures across multiple developmental periods and generation of dose response data. An additional advantage of animal studies is the possibility of conducting *in-vivo* fetal exposure studies in animals which is not possible for human studies which currently rely on *in-vitro* or *ex-viv*o (xenograft) approaches. However, this review has described several exposures for which the studies utilizing human tissues have demonstrated important differences to those found in animals. This may relate to differences in study design, dose administered or exposure regimen; however, it has also been shown that many of these differences appear to result from fundamental species differences in the effect of specific agents at human-relevant levels of exposure. Whilst epidemiological studies can to some extent bridge the gap between effects demonstrated in animal studies and human-relevance, such studies cannot demonstrate direct causation or elicit underlying mechanisms for effects; such studies are also prone to confounding. This highlights the importance of experimental models using human fetal tissues in determining the potential impact of *in-utero* environmental and pharmaceutical exposures in humans.

### Future perspectives

It is clear that understanding effects of *in-utero* exposures on male reproductive development will continue to rely on interpretation of a combination of animal studies, epidemiology and experimental studies utilizing human tissues. Conducting co-ordinated studies that combine these methods represents an important approach. This may include combining experimental studies using *in-vitro* and *in-vivo* approaches (Kristensen *et al.*, 2011; Hurtado-Gonzalez *et al.*, 2018), studies comparing results in both rodent and human tissues (Ben Maamar *et al.*, 2015; Hurtado-Gonzalez *et al.*, 2018), or combined studies of epidemiological and experimental evidence (Kristensen *et al.*, 2011).

Epidemiological studies may be enhanced by developing large-scale prospective cohort studies. This is of particular importance to ensure that the timing of measurement of exposure coincides with the expected mechanism of effect (e.g. *in-utero* exposure and cryptorchidism/hypospadias identified in the neonatal period). Furthermore, for pharmaceuticals with relatively short half-lives, which are taken intermittently and do not accumulate in the body, obtaining accurate and detailed records of exposure during pregnancy is essential. This information is extremely difficult to obtain retrospectively and is prone to recall bias making prospective studies crucial for such exposures.

The future for determining effects of *in-utero* exposure(s) on male testicular development and reproduction is also likely to involve refinement of existing experimental approaches. Whilst recent development of *in-vitro* and xenograft approaches has allowed direct testing of environmental chemicals and pharmaceuticals on the human fetal testis, these models may be limited by tissue supply and heterogeneity between individuals. The recent development of organoids for a variety of organs and tissues may prove critical for future studies to test the effects of exposures in organoids generated from human testicular tissues (Alves-Lopes and Stukenborg, 2017). Computational and mathematical modeling may also be used to predict the effects of exposure(s) *in-silico* (Krysiak-Baltyn *et al.*, 2012), although this will be dependent on the robustness of the imputation of biological data.

The scientific and public interest in the effects of chemical exposures in humans is likely to continue to increase. Regulation of these agents will increasingly rely on models that can provide direct human-relevant data. Therefore, we propose that assessment of the experimental evidence from studies using human fetal tissues should be an integral part of informing regulatory policies in relation to the effects of environmental and pharmaceutical exposures on male reproductive development.

## Supplementary Material

HRU-18-0051-R1-SuppTables_dmz004

## References

[dmz004C1] Abraham M, Alramadhan S, Iniguez C, Duijts L, Jaddoe VW, Den Dekker HT, Crozier S, Godfrey KM, Hindmarsh P, Vik T et al A systematic review of maternal smoking during pregnancy and fetal measurements with meta-analysis. PLoS One 2017;12:e0170946. 28231292 10.1371/journal.pone.0170946 PMC5322900

[dmz004C2] Albert O, Jegou B A critical assessment of the endocrine susceptibility of the human testis to phthalates from fetal life to adulthood. Hum Reprod Update 2014;20:231–249. 24077978 10.1093/humupd/dmt050

[dmz004C3] Alves-Lopes JP, Stukenborg JB Testicular organoids: a new model to study the testicular microenvironment in vitro? Hum Reprod Update 2017;24:176–191. 10.1093/humupd/dmx036 29281008

[dmz004C4] Angenard G, Muczynski V, Coffigny H, Pairault C, Duquenne C, Frydman R, Habert R, Rouiller-Fabre V, Livera G Cadmium increases human fetal germ cell apoptosis. Environ Health Perspect 2010;118:331–337. 20064782 10.1289/ehp.0900975 PMC2854759

[dmz004C5] Axelstad M, Christiansen S, Boberg J, Scholze M, Jacobsen PR, Isling LK, Kortenkamp A, Hass U Mixtures of endocrine-disrupting contaminants induce adverse developmental effects in preweaning rats. Reproduction 2014;147:489–501. 24298046 10.1530/REP-13-0447

[dmz004C6] Ben Maamar M, Lesne L, Desdoits-Lethimonier C, Coiffec I, Lassurguere J, Lavoue V, Deceuninck Y, Antignac JP, Le Bizec B, Perdu E et al An investigation of the endocrine-disruptive effects of bisphenol a in human and rat fetal testes. PLoS One 2015;10:e0117226. 25706302 10.1371/journal.pone.0117226 PMC4338204

[dmz004C7] Ben Maamar M, Lesne L, Hennig K, Desdoits-Lethimonier C, Kilcoyne KR, Coiffec I, Rolland AD, Chevrier C, Kristensen DM, Lavoue V et al Ibuprofen results in alterations of human fetal testis development. Sci Rep 2017;7:44184. 28281692 10.1038/srep44184 PMC5345102

[dmz004C8] Bendsen E, Laursen S, Olesen C, Westergaard L, Andersen C, Byskov A Effect of 4-octylphenol on germ cell number in cultured human fetal gonads. Hum Reprod 2001;16:236–243. 11157813 10.1093/humrep/16.2.236

[dmz004C9] Berkowitz GS, Lapinski RH Risk factors for cryptorchidism: a nested case-control study. Paediatr Perinat Epidemiol 1996;10:39–51. 8746430 10.1111/j.1365-3016.1996.tb00024.x

[dmz004C10] Bitgood MJ, Shen L, McMahon AP Sertoli cell signaling by Desert hedgehog regulates the male germline. Curr Biol 1996;6:298–304. 8805249 10.1016/s0960-9822(02)00480-3

[dmz004C11] Bonde JP, Flachs EM, Rimborg S, Glazer CH, Giwercman A, Ramlau-Hansen CH, Hougaard KS, Hoyer BB, Haervig KK, Petersen SB et al The epidemiologic evidence linking prenatal and postnatal exposure to endocrine disrupting chemicals with male reproductive disorders: a systematic review and meta-analysis. Hum Reprod Update 2016;23:104–125. 27655588 10.1093/humupd/dmw036 PMC5155570

[dmz004C12] Bornehag CG, Carlstedt F, Jonsson BA, Lindh CH, Jensen TK, Bodin A, Jonsson C, Janson S, Swan SH Prenatal phthalate exposures and anogenital distance in Swedish boys. Environ Health Perspect 2015;123:101–107. 25353625 10.1289/ehp.1408163 PMC4286276

[dmz004C13] Carter TC, Druschel CM, Romitti PA, Bell EM, Werler MM, Mitchell AA National Birth Defects Prevention S. Antifungal drugs and the risk of selected birth defects. Am J Obstet Gynecol 2008;198:191 e191–e197. 18226621 10.1016/j.ajog.2007.08.044

[dmz004C14] Chauvigne F, Menuet A, Lesne L, Chagnon MC, Chevrier C, Regnier JF, Angerer J, Jegou B Time- and dose-related effects of di-(2-ethylhexyl) phthalate and its main metabolites on the function of the rat fetal testis in vitro. Environ Health Perspect 2009;117:515–521. 19440488 10.1289/ehp.11870 PMC2679593

[dmz004C15] Chevalier N, Brucker-Davis F, Lahlou N, Coquillard P, Pugeat M, Pacini P, Panaia-Ferrari P, Wagner-Mahler K, Fenichel P A negative correlation between insulin-like peptide 3 and bisphenol A in human cord blood suggests an effect of endocrine disruptors on testicular descent during fetal development. Hum Reprod 2015;30:447–453. 25527819 10.1093/humrep/deu340

[dmz004C16] Chevrier C, Petit C, Philippat C, Mortamais M, Slama R, Rouget F, Calafat AM, Ye X, Silva MJ, Charles MA et al Maternal urinary phthalates and phenols and male genital anomalies. Epidemiology 2012;23:353–356. 22317818 10.1097/EDE.0b013e318246073e PMC4724202

[dmz004C17] Coutts SM, Fulton N, Anderson RA Environmental toxicant-induced germ cell apoptosis in the human fetal testis. Hum Reprod 2007;22:2912–2918. 17890726 10.1093/humrep/dem300

[dmz004C18] Damgaard IN, Jensen TK, Nordic Cryptorchidism Study G, Petersen JH, Skakkebaek NE, Toppari J, Main KM Risk factors for congenital cryptorchidism in a prospective birth cohort study. PLoS One 2008;3:e3051. 18725961 10.1371/journal.pone.0003051 PMC2516600

[dmz004C19] Damgaard IN, Jensen TK, Petersen JH, Skakkebaek NE, Toppari J, Main KM Cryptorchidism and maternal alcohol consumption during pregnancy. Environ Health Perspect 2007;115:272–277. 17384777 10.1289/ehp.9608 PMC1817679

[dmz004C20] Damgaard IN, Skakkebaek NE, Toppari J, Virtanen HE, Shen H, Schramm KW, Petersen JH, Jensen TK, Main KM, Nordic Cryptorchidism Study G Persistent pesticides in human breast milk and cryptorchidism. Environ Health Perspect 2006;114:1133–1138. 16835070 10.1289/ehp.8741 PMC1513324

[dmz004C21] Dean A, Mungall W, McKinnell C, Sharpe RM Prostaglandins, masculinization and its disorders: effects of fetal exposure of the rat to the cyclooxygenase inhibitor-indomethacin. PLoS One 2013;8:e62556. 23671609 10.1371/journal.pone.0062556 PMC3643956

[dmz004C22] Dean A, Sharpe RM Clinical review: anogenital distance or digit length ratio as measures of fetal androgen exposure: relationship to male reproductive development and its disorders. J Clin Endocrinol Metab 2013;98:2230–2238. 23569219 10.1210/jc.2012-4057

[dmz004C23] Delbes G, Levacher C, Duquenne C, Racine C, Pakarinen P, Habert R Endogenous estrogens inhibit mouse fetal Leydig cell development via estrogen receptor alpha. Endocrinology 2005;146:2454–2461. 15661855 10.1210/en.2004-1540

[dmz004C24] Dere E, Anderson LM, Huse SM, Spade DJ, McDonnell-Clark E, Madnick SJ, Hall SJ, Camacho L, Lewis SM, Vanlandingham MM et al Effects of continuous bisphenol A exposure from early gestation on 90 day old rat testes function and sperm molecular profiles: a CLARITY-BPA consortium study. Toxicol Appl Pharmacol 2018;347:1–9. 29596923 10.1016/j.taap.2018.03.021 PMC6412024

[dmz004C25] Eladak S, Grisin T, Moison D, Guerquin MJ, N’Tumba-Byn T, Pozzi-Gaudin S, Benachi A, Livera G, Rouiller-Fabre V, Habert R A new chapter in the bisphenol A story: bisphenol S and bisphenol F are not safe alternatives to this compound. Fertil Steril 2015;103:11–21. 25475787 10.1016/j.fertnstert.2014.11.005

[dmz004C26] Eladak S, Moison D, Guerquin MJ, Matilionyte G, Kilcoyne K, N’Tumba-Byn T, Messiaen S, Deceuninck Y, Pozzi-Gaudin S, Benachi A et al Effects of environmental bisphenol A exposures on germ cell development and Leydig cell function in the human fetal testis. PLoS One 2018;13:e0191934. 29385186 10.1371/journal.pone.0191934 PMC5791995

[dmz004C27] Fenichel P, Dechaux H, Harthe C, Gal J, Ferrari P, Pacini P, Wagner-Mahler K, Pugeat M, Brucker-Davis F Unconjugated bisphenol A cord blood levels in boys with descended or undescended testes. Hum Reprod 2012;27:983–990. 22267833 10.1093/humrep/der451

[dmz004C28] Fernandez MF, Arrebola JP, Jimenez-Diaz I, Saenz JM, Molina-Molina JM, Ballesteros O, Kortenkamp A, Olea N Bisphenol A and other phenols in human placenta from children with cryptorchidism or hypospadias. Reprod Toxicol 2016;59:89–95. 26602963 10.1016/j.reprotox.2015.11.002

[dmz004C29] Ferrara D, Hallmark N, Scott H, Brown R, McKinnell C, Mahood IK, Sharpe RM Acute and long-term effects of in utero exposure of rats to di(n-butyl) phthalate on testicular germ cell development and proliferation. Endocrinology 2006;147:5352–5362. 16916955 10.1210/en.2006-0527

[dmz004C30] Fisher BG, Thankamony A, Hughes IA, Ong KK, Dunger DB, Acerini CL Prenatal paracetamol exposure is associated with shorter anogenital distance in male infants. Hum Reprod 2016;31:2642–2650. 27609981 10.1093/humrep/dew196 PMC5088633

[dmz004C31] Foster WG, Evans JA, Little J, Arbour L, Moore A, Sauve R, Andres Leon J, Luo W Human exposure to environmental contaminants and congenital anomalies: a critical review. Crit Rev Toxicol 2017;47:59–84. 27685638 10.1080/10408444.2016.1211090

[dmz004C32] Fowler PA, Abramovich DR, Haites NE, Cash P, Groome NP, Al-Qahtani A, Murray TJ, Lea RG Human fetal testis Leydig cell disruption by exposure to the pesticide dieldrin at low concentrations. Hum Reprod 2007;22:2919–2927. 17848404 10.1093/humrep/dem256

[dmz004C33] Fowler PA, Bhattacharya S, Flannigan S, Drake AJ, O’Shaughnessy PJ Maternal cigarette smoking and effects on androgen action in male offspring: unexpected effects on second-trimester anogenital distance. J Clin Endocrinol Metab 2011;96:E1502–E1506. 21752894 10.1210/jc.2011-1100

[dmz004C34] Fowler PA, Bhattacharya S, Gromoll J, Monteiro A, O’Shaughnessy PJ Maternal smoking and developmental changes in luteinizing hormone (LH) and the LH receptor in the fetal testis. J Clin Endocrinol Metab 2009;94:4688–4695. 19837924 10.1210/jc.2009-0994 PMC2848822

[dmz004C35] Gaudriault P, Mazaud-Guittot S, Lavoue V, Coiffec I, Lesne L, Dejucq-Rainsford N, Scholze M, Kortenkamp A, Jegou B Endocrine disruption in human fetal testis explants by individual and combined exposures to selected pharmaceuticals, pesticides, and environmental pollutants. Environ Health Perspect 2017;125:087004. 28796631 10.1289/EHP1014 PMC5783658

[dmz004C36] Gill WB, Schumacher GF, Bibbo M, Straus FH 2nd, Schoenberg HW Association of diethylstilbestrol exposure in utero with cryptorchidism, testicular hypoplasia and semen abnormalities. J Urol 1979;122:36–39. 37351 10.1016/s0022-5347(17)56240-0

[dmz004C37] Goen T, Dobler L, Koschorreck J, Muller J, Wiesmuller GA, Drexler H, Kolossa-Gehring M Trends of the internal phthalate exposure of young adults in Germany—follow-up of a retrospective human biomonitoring study. Int J Hyg Environ Health 2011;215:36–45. 21889907 10.1016/j.ijheh.2011.07.011

[dmz004C38] Gray LE Jr., Ostby J, Furr J, Price M, Veeramachaneni DN, Parks L Perinatal exposure to the phthalates DEHP, BBP, and DINP, but not DEP, DMP, or DOTP, alters sexual differentiation of the male rat. Toxicol Sci 2000;58:350–365. 11099647 10.1093/toxsci/58.2.350

[dmz004C39] Griswold SL, Behringer RR Fetal Leydig cell origin and development. Sex Dev 2009;3:1–15. 19339813 10.1159/000200077 PMC4021856

[dmz004C40] Gupta C, Goldman AS The arachidonic acid cascade is involved in the masculinizing action of testosterone on embryonic external genitalia in mice. Proc Natl Acad Sci USA 1986;83:4346–4349. 3086881 10.1073/pnas.83.12.4346 PMC323729

[dmz004C41] Haavisto T, Nurmela K, Pohjanvirta R, Huuskonen H, El-Gehani F, Paranko J Prenatal testosterone and luteinizing hormone levels in male rats exposed during pregnancy to 2,3,7,8-tetrachlorodibenzo-p-dioxin and diethylstilbestrol. Mol Cell Endocrinol 2001;178:169–179. 11403907 10.1016/s0303-7207(01)00425-7

[dmz004C42] Habert R, Livera G, Rouiller-Fabre V Man is not a big rat: concerns with traditional human risk assessment of phthalates based on their anti-androgenic effects observed in the rat foetus. Basic Clin Androl 2014;24:14. 25780587 10.1186/2051-4190-24-14 PMC4349750

[dmz004C43] Hait EJ, Calafat AM, Hauser R Urinary phthalate metabolite concentrations among men with inflammatory bowel disease on mesalamine therapy. Endocr Disruptors (Austin) 2014;1:e25066. 25392847 10.4161/endo.25066 PMC4226411

[dmz004C44] Hallmark N, Walker M, McKinnell C, Mahood IK, Scott H, Bayne R, Coutts S, Anderson RA, Greig I, Morris K et al Effects of monobutyl and di(n-butyl) phthalate in vitro on steroidogenesis and Leydig cell aggregation in fetal testis explants from the rat: comparison with effects in vivo in the fetal rat and neonatal marmoset and in vitro in the human. Environ Health Perspect 2007;115:390–396. 17431488 10.1289/ehp.9490 PMC1849934

[dmz004C45] Heeren AM, van Iperen L, Klootwijk DB, de Melo Bernardo A, Roost MS, Gomes Fernandes MM, Louwe LA, Hilders CG, Helmerhorst FM, van der Westerlaken LA et al Development of the follicular basement membrane during human gametogenesis and early folliculogenesis. BMC Dev Biol 2015;15:4. 25605128 10.1186/s12861-015-0054-0 PMC4307144

[dmz004C46] Heger NE, Hall SJ, Sandrof MA, McDonnell EV, Hensley JB, McDowel EN, Martin KA, Gaido KW, Johnson KJ, Boekelheide K Human fetal testis xenografts are resistant to phthalate-induced endocrine disruption. Environ Health Perspect 2012;120:1137–1143. 22511013 10.1289/ehp.1104711 PMC3440087

[dmz004C47] Henderson BE, Benton B, Cosgrove M, Baptista J, Aldrich J, Townsend D, Hart W, Mack TM Urogenital tract abnormalities in sons of women treated with diethylstilbestrol. Pediatrics 1976;58:505–507. 972792

[dmz004C48] Herbst AL, Ulfelder H, Poskanzer DC Adenocarcinoma of the vagina. Association of maternal stilbestrol therapy with tumor appearance in young women. N Engl J Med 1971;284:878–881. 5549830 10.1056/NEJM197104222841604

[dmz004C49] Hernandez-Diaz S, Mitchell AA, Kelley KE, Calafat AM, Hauser R Medications as a potential source of exposure to phthalates in the U.S. population. Environ Health Perspect 2009;117:185–189. 19270786 10.1289/ehp.11766 PMC2649218

[dmz004C50] Holm JB, Chalmey C, Modick H, Jensen LS, Dierkes G, Weiss T, Jensen BA, Norregard MM, Borkowski K, Styrishave B et al Aniline is rapidly converted into paracetamol impairing male reproductive development. Toxicol Sci 2015;148:288–298. 26259604 10.1093/toxsci/kfv179

[dmz004C51] Howdeshell KL, Furr J, Lambright CR, Wilson VS, Ryan BC, Gray LE Jr Gestational and lactational exposure to ethinyl estradiol, but not bisphenol A, decreases androgen-dependent reproductive organ weights and epididymal sperm abundance in the male long evans hooded rat. Toxicol Sci 2008;102:371–382. 18096570 10.1093/toxsci/kfm306

[dmz004C52] Huang PC, Kuo PL, Chou YY, Lin SJ, Lee CC Association between prenatal exposure to phthalates and the health of newborns. Environ Int 2009;35:14–20. 18640725 10.1016/j.envint.2008.05.012

[dmz004C53] Hughes IA, Acerini CL Factors controlling testis descent. Eur J Endocrinol 2008;159:S75–S82. 18647820 10.1530/EJE-08-0458

[dmz004C54] Hurtado-Gonzalez P, Anderson RA, Macdonald J, van den Driesche S, Kilcoyne K, Jorgensen A, McKinnell C, Macpherson S, Sharpe RM, Mitchell RT Effects of exposure to acetaminophen and ibuprofen on fetal germ cell development in both sexes in rodent and human using multiple experimental systems. Environ Health Perspect 2018;126:047006. 29665328 10.1289/EHP2307 PMC6071829

[dmz004C55] Hurtado-Gonzalez P, Mitchell RT Analgesic use in pregnancy and male reproductive development. Curr Opin Endocrinol Diabetes Obes 2017;24:225–232. 28277341 10.1097/MED.0000000000000338 PMC5423522

[dmz004C56] Jamieson L, McCully W Review: UK medicines likely to be affected by the proposed European Medicines Agency’s guidelines on phthalates. BMC Pharmacol Toxicol 2015;16:17. 26070463 10.1186/s40360-015-0018-9 PMC4465162

[dmz004C57] Jensen MS, Anand-Ivell R, Norgaard-Pedersen B, Jonsson BA, Bonde JP, Hougaard DM, Cohen A, Lindh CH, Ivell R, Toft G Amniotic fluid phthalate levels and male fetal gonad function. Epidemiology 2015;26:91–99. 25384265 10.1097/EDE.0000000000000198

[dmz004C58] Jensen TK, Frederiksen H, Kyhl HB, Lassen TH, Swan SH, Bornehag CG, Skakkebaek NE, Main KM, Lind DV, Husby S et al Prenatal exposure to phthalates and anogenital distance in male infants from a low-exposed Danish cohort (2010-2012). Environ Health Perspect 2016;124:1107–1113. 26672060 10.1289/ehp.1509870 PMC4937858

[dmz004C59] Jensen MS, Rebordosa C, Thulstrup AM, Toft G, Sorensen HT, Bonde JP, Henriksen TB, Olsen J Maternal use of acetaminophen, ibuprofen, and acetylsalicylic acid during pregnancy and risk of cryptorchidism. Epidemiology 2010;21:779–785. 20805751 10.1097/EDE.0b013e3181f20bed

[dmz004C60] Jensen MS, Toft G, Thulstrup AM, Bonde JP, Olsen J Cryptorchidism according to maternal gestational smoking. Epidemiology 2007;18:220–225. 17202869 10.1097/01.ede.0000254061.90686.9f

[dmz004C61] Jobling MS, Hutchison GR, van den Driesche S, Sharpe RM Effects of di(n-butyl) phthalate exposure on foetal rat germ-cell number and differentiation: identification of age-specific windows of vulnerability. Int J Androl 2011;34:e386–e396. 21332505 10.1111/j.1365-2605.2010.01140.x PMC3229675

[dmz004C62] Joffe M Myths about endocrine disruption and the male reproductive system should not be propagated. Hum Reprod 2002;17:520–523. 11821308 10.1093/humrep/17.2.520

[dmz004C63] Kelley KE, Hernandez-Diaz S, Chaplin EL, Hauser R, Mitchell AA Identification of phthalates in medications and dietary supplement formulations in the United States and Canada. Environ Health Perspect 2012;120:379–384. 22169271 10.1289/ehp.1103998 PMC3295354

[dmz004C64] Kilcoyne KR, Mitchell RT Assessing the impact of in-utero exposures: potential effects of paracetamol on male reproductive development. Arch Dis Child 2017;102:1169–1175. 28588045 10.1136/archdischild-2016-311374

[dmz004C65] Kilcoyne KR, Smith LB, Atanassova N, Macpherson S, McKinnell C, van den Driesche S, Jobling MS, Chambers TJ, De Gendt K, Verhoeven G et al Fetal programming of adult Leydig cell function by androgenic effects on stem/progenitor cells. Proc Natl Acad Sci USA 2014;111:E1924–E1932. 24753613 10.1073/pnas.1320735111 PMC4020050

[dmz004C66] Kobayashi K, Miyagawa M, Wang RS, Sekiguchi S, Suda M, Honma T Effects of in utero and lactational exposure to bisphenol A on somatic growth and anogenital distance in F1 rat offspring. Ind Health 2002;40:375–381. 12502241 10.2486/indhealth.40.375

[dmz004C67] Koch HM, Christensen KL, Harth V, Lorber M, Bruning T Di-n-butyl phthalate (DnBP) and diisobutyl phthalate (DiBP) metabolism in a human volunteer after single oral doses. Arch Toxicol 2012;86:1829–1839. 22820759 10.1007/s00204-012-0908-1

[dmz004C68] Komarowska MD, Hermanowicz A, Czyzewska U, Milewski R, Matuszczak E, Miltyk W, Debek W Serum bisphenol A level in boys with cryptorchidism: a step to male infertility? Int J Endocrinol 2015;2015:973154. 26491444 10.1155/2015/973154 PMC4600910

[dmz004C69] Kristensen DM, Hass U, Lesne L, Lottrup G, Jacobsen PR, Desdoits-Lethimonier C, Boberg J, Petersen JH, Toppari J, Jensen TK et al Intrauterine exposure to mild analgesics is a risk factor for development of male reproductive disorders in human and rat. Hum Reprod 2011;26:235–244. 21059752 10.1093/humrep/deq323

[dmz004C70] Kristensen DM, Lesne L, Le Fol V, Desdoits-Lethimonier C, Dejucq-Rainsford N, Leffers H, Jegou B Paracetamol (acetaminophen), aspirin (acetylsalicylic acid) and indomethacin are anti-androgenic in the rat foetal testis. Int J Androl 2012;35:377–384. 22612476 10.1111/j.1365-2605.2012.01282.x

[dmz004C71] Kristensen DM, Mazaud-Guittot S, Gaudriault P, Lesne L, Serrano T, Main KM, Jegou B Analgesic use—prevalence, biomonitoring and endocrine and reproductive effects. Nat Rev Endocrinol 2016;12:381–393. 27150289 10.1038/nrendo.2016.55

[dmz004C72] Krysiak-Baltyn K, Toppari J, Skakkebaek NE, Jensen TS, Virtanen HE, Schramm KW, Shen H, Vartiainen T, Kiviranta H, Taboureau O et al Association between chemical pattern in breast milk and congenital cryptorchidism: modelling of complex human exposures. Int J Androl 2012;35:294–302. 22519522 10.1111/j.1365-2605.2012.01268.x

[dmz004C73] Lambrot R, Muczynski V, Lecureuil C, Angenard G, Coffigny H, Pairault C, Moison D, Frydman R, Habert R, Rouiller-Fabre V Phthalates impair germ cell development in the human fetal testis in vitro without change in testosterone production. Environ Health Perspect 2009;117:32–37. 19165384 10.1289/ehp.11146 PMC2627862

[dmz004C74] Latini G, De Felice C, Presta G, Del Vecchio A, Paris I, Ruggieri F, Mazzeo P In utero exposure to di-(2-ethylhexyl)phthalate and duration of human pregnancy. Environ Health Perspect 2003;111:1783–1785. 14594632 10.1289/ehp.6202 PMC1241724

[dmz004C75] Leary FJ, Resseguie LJ, Kurland LT, O’Brien PC, Emslander RF, Noller KL Males exposed in utero to diethylstilbestrol. J Am Med Assoc 1984;252:2984–2989. 6502859

[dmz004C76] Leffers H, Naesby M, Vendelbo B, Skakkebaek NE, Jorgensen M Oestrogenic potencies of Zeranol, oestradiol, diethylstilboestrol, Bisphenol-A and genistein: implications for exposure assessment of potential endocrine disrupters. Hum Reprod 2001;16:1037–1045. 11331657 10.1093/humrep/16.5.1037

[dmz004C77] Lehraiki A, Racine C, Krust A, Habert R, Levacher C Phthalates impair germ cell number in the mouse fetal testis by an androgen- and estrogen-independent mechanism. Toxicol Sci 2009;111:372–382. 19592451 10.1093/toxsci/kfp153 PMC2742583

[dmz004C78] Li H, Kim KH Effects of mono-(2-ethylhexyl) phthalate on fetal and neonatal rat testis organ cultures. Biol Reprod 2003;69:1964–1972. 12904314 10.1095/biolreprod.103.018895

[dmz004C79] Lin LC, Wang SL, Chang YC, Huang PC, Cheng JT, Su PH, Liao PC Associations between maternal phthalate exposure and cord sex hormones in human infants. Chemosphere 2011;83:1192–1199. 21272909 10.1016/j.chemosphere.2010.12.079

[dmz004C80] Main KM, Mortensen GK, Kaleva MM, Boisen KA, Damgaard IN, Chellakooty M, Schmidt IM, Suomi AM, Virtanen HE, Petersen DV et al Human breast milk contamination with phthalates and alterations of endogenous reproductive hormones in infants three months of age. Environ Health Perspect 2006;114:270–276. 16451866 10.1289/ehp.8075 PMC1367843

[dmz004C81] Marsee K, Woodruff TJ, Axelrad DA, Calafat AM, Swan SH Estimated daily phthalate exposures in a population of mothers of male infants exhibiting reduced anogenital distance. Environ Health Perspect 2006;114:805–809. 16759976 10.1289/ehp.8663 PMC1480516

[dmz004C82] Marselos M, Tomatis L Diethylstilboestrol: I, Pharmacology, toxicology and carcinogenicity in humans. Eur J Cancer 1992;28A:1182–1189. 1627392 10.1016/0959-8049(92)90482-h

[dmz004C83] Martin OV, Shialis T, Lester JN, Scrimshaw MD, Boobis AR, Voulvoulis N Testicular dysgenesis syndrome and the estrogen hypothesis: a quantitative meta-analysis. Environ Health Perspect 2008;116:149–157. 18288311 10.1289/ehp.10545 PMC2235228

[dmz004C84] Martino-Andrade AJ, Liu F, Sathyanarayana S, Barrett ES, Redmon JB, Nguyen RH, Levine H, Swan SH, Team TS Timing of prenatal phthalate exposure in relation to genital endpoints in male newborns. Andrology 2016;4:585–593. 27062102 10.1111/andr.12180

[dmz004C85] Mazaud-Guittot S, Nicolas Nicolaz C, Desdoits-Lethimonier C, Coiffec I, Ben Maamar M, Balaguer P, Kristensen DM, Chevrier C, Lavoue V, Poulain P et al Paracetamol, aspirin, and indomethacin induce endocrine disturbances in the human fetal testis capable of interfering with testicular descent. J Clin Endocrinol Metab 2013;98:E1757–E1767. 24030937 10.1210/jc.2013-2531

[dmz004C86] McKinnell C, Mitchell RT, Morris K, Anderson RA, Kelnar CJ, Wallace WH, Sharpe RM Perinatal germ cell development and differentiation in the male marmoset (Callithrix jacchus): similarities with the human and differences from the rat. Hum Reprod 2013;28:886–896. 23321215 10.1093/humrep/des465 PMC3600838

[dmz004C87] McKinnell C, Mitchell RT, Walker M, Morris K, Kelnar CJ, Wallace WH, Sharpe RM Effect of fetal or neonatal exposure to monobutyl phthalate (MBP) on testicular development and function in the marmoset. Hum Reprod 2009;24:2244–2254. 19491204 10.1093/humrep/dep200 PMC2727403

[dmz004C88] Miao M, Yuan W, He Y, Zhou Z, Wang J, Gao E, Li G, Li DK In utero exposure to bisphenol-A and anogenital distance of male offspring. Birth Defects Res A Clin Mol Teratol 2011;91:867–872. 21987463 10.1002/bdra.22845

[dmz004C89] Mitchell RT, Childs AJ, Anderson RA, van den Driesche S, Saunders PTK, McKinnell C, Wallace WHB, Kelnar CJH, Sharpe RM Do Phthalates affect steroidogenesis by the human fetal testis? Exposure of human fetal testis xenografts to di-n-butyl phthalate. Journal of Clinical Endocrinology & Metabolism 2012;97:E341–E348. 22238399 10.1210/jc.2011-2411

[dmz004C90] Mitchell RT, Cowan G, Morris KD, Anderson RA, Fraser HM, McKenzie KJ, Wallace WH, Kelnar CJ, Saunders PT, Sharpe RM Germ cell differentiation in the marmoset (Callithrix jacchus) during fetal and neonatal life closely parallels that in the human. Hum Reprod 2008;23:2755–2765. 18694875 10.1093/humrep/den295 PMC2583943

[dmz004C91] Mitchell RT, Saunders PT, Childs AJ, Cassidy-Kojima C, Anderson RA, Wallace WH, Kelnar CJ, Sharpe RM Xenografting of human fetal testis tissue: a new approach to study fetal testis development and germ cell differentiation. Hum Reprod 2010;25:2405–2414. 20683063 10.1093/humrep/deq183 PMC2939754

[dmz004C92] Mitchell RT, Sharpe RM, Anderson RA, McKinnell C, Macpherson S, Smith LB, Wallace WH, Kelnar CJ, van den Driesche S Diethylstilboestrol exposure does not reduce testosterone production in human fetal testis xenografts. PLoS One 2013;8:e61726. 23620786 10.1371/journal.pone.0061726 PMC3631175

[dmz004C93] Modick H, Weiss T, Dierkes G, Bruning T, Koch HM Ubiquitous presence of paracetamol in human urine: sources and implications. Reproduction 2014;147:R105–R117. 24451225 10.1530/REP-13-0527

[dmz004C94] Modick H, Weiss T, Dierkes G, Koslitz S, Kafferlein HU, Bruning T, Koch HM Human metabolism and excretion kinetics of aniline after a single oral dose. Arch Toxicol 2016;90:1325–1333. 26233686 10.1007/s00204-015-1566-x

[dmz004C95] Mogensen DM, Pihl MB, Skakkebaek NE, Andersen HR, Juul A, Kyhl HB, Swan S, Kristensen DM, Andersen MS, Lind DV et al Prenatal exposure to antifungal medication may change anogenital distance in male offspring: a preliminary study. Environ Health 2017;16:68. 28637461 10.1186/s12940-017-0263-z PMC5480178

[dmz004C96] Moher D, Liberati A, Tetzlaff J, Altman DG, Group P Preferred reporting items for systematic reviews and meta-analyses: the PRISMA statement. PLoS Med 2009;6:e1000097. 19621072 10.1371/journal.pmed.1000097 PMC2707599

[dmz004C97] Muczynski V, Cravedi JP, Lehraiki A, Levacher C, Moison D, Lecureuil C, Messiaen S, Perdu E, Frydman R, Habert R et al Effect of mono-(2-ethylhexyl) phthalate on human and mouse fetal testis: In vitro and in vivo approaches. Toxicol Appl Pharmacol 2012a;261:97–104. 22484159 10.1016/j.taap.2012.03.016

[dmz004C98] Muczynski V, Lecureuil C, Messiaen S, Guerquin MJ, N’Tumba-Byn T, Moison D, Hodroj W, Benjelloun H, Baijer J, Livera G et al Cellular and molecular effect of MEHP Involving LXRalpha in human fetal testis and ovary. PLoS One 2012b;7:e48266. 23118965 10.1371/journal.pone.0048266 PMC3484128

[dmz004C99] Mylchreest E, Sar M, Wallace DG, Foster PM Fetal testosterone insufficiency and abnormal proliferation of Leydig cells and gonocytes in rats exposed to di(n-butyl) phthalate. Reprod Toxicol 2002;16:19–28. 11934529 10.1016/s0890-6238(01)00201-5

[dmz004C100] Nash A, Davies L Fetal alcohol spectrum disorders: what pediatric providers need to know. J Pediatr Health Care 2017;31:594–606. 28838601 10.1016/j.pedhc.2017.04.002

[dmz004C101] Norgaard M, Pedersen L, Gislum M, Erichsen R, Sogaard KK, Schonheyder HC, Sorensen HT Maternal use of fluconazole and risk of congenital malformations: a Danish population-based cohort study. J Antimicrob Chemother 2008;62:172–176. 18400803 10.1093/jac/dkn157

[dmz004C102] N’Tumba-Byn T, Moison D, Lacroix M, Lecureuil C, Lesage L, Prud’homme SM, Pozzi-Gaudin S, Frydman R, Benachi A, Livera G et al Differential effects of bisphenol A and diethylstilbestrol on human, rat and mouse fetal leydig cell function. PLoS One 2012;7:e51579. 23284716 10.1371/journal.pone.0051579 PMC3524173

[dmz004C103] Ostby J, Kelce WR, Lambright C, Wolf CJ, Mann P, Gray LE Jr. The fungicide procymidone alters sexual differentiation in the male rat by acting as an androgen-receptor antagonist in vivo and in vitro. Toxicol Ind Health 1999;15:80–93. 10188193 10.1177/074823379901500108

[dmz004C104] O’Shaughnessy PJ, Fowler PA Endocrinology of the mammalian fetal testis. Reproduction 2011;141:37–46. 20956578 10.1530/REP-10-0365

[dmz004C105] Palmer JR, Herbst AL, Noller KL, Boggs DA, Troisi R, Titus-Ernstoff L, Hatch EE, Wise LA, Strohsnitter WC, Hoover RN Urogenital abnormalities in men exposed to diethylstilbestrol in utero: a cohort study. Environ Health 2009;8:37. 19689815 10.1186/1476-069X-8-37 PMC2739506

[dmz004C106] Parks LG, Ostby JS, Lambright CR, Abbott BD, Klinefelter GR, Barlow NJ, Gray LE Jr. The plasticizer diethylhexyl phthalate induces malformations by decreasing fetal testosterone synthesis during sexual differentiation in the male rat. Toxicol Sci 2000;58:339–349. 11099646 10.1093/toxsci/58.2.339

[dmz004C107] Philippat C, Giorgis-Allemand L, Chevrier C, Cordier S, Jegou B, Charles MA, Slama R Analgesics during pregnancy and undescended testis. Epidemiology 2011;22:747–749. 21811116 10.1097/EDE.0b013e318225bf33

[dmz004C108] Pierucci-Alves F, Clark AM, Russell LD A developmental study of the Desert hedgehog-null mouse testis. Biol Reprod 2001;65:1392–1402. 11673255 10.1095/biolreprod65.5.1392

[dmz004C109] Rahimi R, Nikfar S, Rezaie A, Abdollahi M Pregnancy outcome in women with inflammatory bowel disease following exposure to 5-aminosalicylic acid drugs: a meta-analysis. Reprod Toxicol 2008;25:271–275. 18242053 10.1016/j.reprotox.2007.11.010

[dmz004C110] Rajpert-De Meyts E, McGlynn KA, Okamoto K, Jewett MA, Bokemeyer C Testicular germ cell tumours. Lancet 2016;387:1762–1774. 26651223 10.1016/S0140-6736(15)00991-5

[dmz004C111] Ramlau-Hansen CH, Thulstrup AM, Storgaard L, Toft G, Olsen J, Bonde JP Is prenatal exposure to tobacco smoking a cause of poor semen quality? A follow-up study. Am J Epidemiol 2007;165:1372–1379. 17369608 10.1093/aje/kwm032

[dmz004C112] Rayburn W, Shukla U, Stetson P, Piehl E Acetaminophen pharmacokinetics: comparison between pregnant and nonpregnant women. Am J Obstet Gynecol 1986;155:1353–1356. 3789044 10.1016/0002-9378(86)90173-0

[dmz004C113] Robert F, Fendri S, Hary L, Lacroix C, Andrejak M, Lalau JD Kinetics of plasma and erythrocyte metformin after acute administration in healthy subjects. Diabetes Metab 2003;29:279–283. 12909816 10.1016/s1262-3636(07)70037-x

[dmz004C114] Robitaille CN, Rivest P, Sanderson JT Antiandrogenic mechanisms of pesticides in human LNCaP prostate and H295R adrenocortical carcinoma cells. Toxicol Sci 2015;143:126–135. 25324206 10.1093/toxsci/kfu212

[dmz004C115] Schug TT, Heindel JJ, Camacho L, Delclos KB, Howard P, Johnson AF, Aungst J, Keefe D, Newbold R, Walker NJ et al A new approach to synergize academic and guideline-compliant research: the CLARITY-BPA research program. Reprod Toxicol 2013;40:35–40. 23747832 10.1016/j.reprotox.2013.05.010

[dmz004C116] Scott HM, Mason JI, Sharpe RM Steroidogenesis in the fetal testis and its susceptibility to disruption by exogenous compounds. Endocr Rev 2009;30:883–925. 19887492 10.1210/er.2009-0016

[dmz004C117] Seckin E, Fromme H, Volkel W Determination of total and free mono-n-butyl phthalate in human urine samples after medication of a di-n-butyl phthalate containing capsule. Toxicol Lett 2009;188:33–37. 19433267 10.1016/j.toxlet.2009.03.002

[dmz004C118] Sharpe RM, Skakkebaek NE Testicular dysgenesis syndrome: mechanistic insights and potential new downstream effects. Fertil Steril 2008;89:e33–e38. 18308057 10.1016/j.fertnstert.2007.12.026

[dmz004C119] Shen H, Main KM, Andersson AM, Damgaard IN, Virtanen HE, Skakkebaek NE, Toppari J, Schramm KW Concentrations of persistent organochlorine compounds in human milk and placenta are higher in Denmark than in Finland. Hum Reprod 2008;23:201–210. 18025027 10.1093/humrep/dem199

[dmz004C120] Silva MJ, Jia T, Samandar E, Preau JL Jr., Calafat AM Environmental exposure to the plasticizer 1,2-cyclohexane dicarboxylic acid, diisononyl ester (DINCH) in U.S. adults (2000-2012). Environ Res 2013;126:159–163. 23777640 10.1016/j.envres.2013.05.007 PMC4554753

[dmz004C121] Skakkebaek NE, Rajpert-De Meyts E, Buck Louis GM, Toppari J, Andersson AM, Eisenberg ML, Jensen TK, Jorgensen N, Swan SH, Sapra KJ et al Male reproductive disorders and fertility trends: influences of environment and genetic susceptibility. Physiol Rev 2016;96:55–97. 26582516 10.1152/physrev.00017.2015 PMC4698396

[dmz004C122] Snijder CA, Kortenkamp A, Steegers EA, Jaddoe VW, Hofman A, Hass U, Burdorf A Intrauterine exposure to mild analgesics during pregnancy and the occurrence of cryptorchidism and hypospadia in the offspring: the Generation R Study. Hum Reprod 2012;27:1191–1201. 22301570 10.1093/humrep/der474

[dmz004C123] Spade DJ, Hall SJ, Saffarini CM, Huse SM, McDonnell EV, Boekelheide K Differential response to abiraterone acetate and di-n-butyl phthalate in an androgen-sensitive human fetal testis xenograft bioassay. Toxicol Sci 2014;138:148–160. 24284787 10.1093/toxsci/kft266 PMC3930360

[dmz004C124] Storgaard L, Bonde JP, Ernst E, Spano M, Andersen CY, Frydenberg M, Olsen J Does smoking during pregnancy affect sons’ sperm counts? Epidemiology 2003;14:278–286. 12859027

[dmz004C125] Storgaard L, Bonde JP, Olsen J Male reproductive disorders in humans and prenatal indicators of estrogen exposure. A review of published epidemiological studies. Reprod Toxicol 2006;21:4–15. 16005180 10.1016/j.reprotox.2005.05.006

[dmz004C126] Suzuki Y, Yoshinaga J, Mizumoto Y, Serizawa S, Shiraishi H Foetal exposure to phthalate esters and anogenital distance in male newborns. Int J Androl 2012;35:236–244. 21696396 10.1111/j.1365-2605.2011.01190.x

[dmz004C127] Swan SH Environmental phthalate exposure in relation to reproductive outcomes and other health endpoints in humans. Environ Res 2008;108:177–184. 18949837 10.1016/j.envres.2008.08.007 PMC2775531

[dmz004C128] Swan SH, Main KM, Liu F, Stewart SL, Kruse RL, Calafat AM, Mao CS, Redmon JB, Ternand CL, Sullivan S et al Decrease in anogenital distance among male infants with prenatal phthalate exposure. Environ Health Perspect 2005;113:1056–1061. 16079079 10.1289/ehp.8100 PMC1280349

[dmz004C129] Swan SH, Sathyanarayana S, Barrett ES, Janssen S, Liu F, Nguyen RH, Redmon JB, Team TS First trimester phthalate exposure and anogenital distance in newborns. Hum Reprod 2015;30:963–972. 25697839 10.1093/humrep/deu363 PMC4359397

[dmz004C130] Tanaka M, Nakaya S, Katayama M, Leffers H, Nozawa S, Nakazawa R, Iwamoto T, Kobayashi S Effect of prenatal exposure to bisphenol A on the serum testosterone concentration of rats at birth. Hum Exp Toxicol 2006;25:369–373. 16898165 10.1191/0960327106ht638oa

[dmz004C131] Tartarin P, Moison D, Guibert E, Dupont J, Habert R, Rouiller-Fabre V, Frydman N, Pozzi S, Frydman R, Lecureuil C et al Metformin exposure affects human and mouse fetal testicular cells. Hum Reprod 2012;27:3304–3314. 22811314 10.1093/humrep/des264

[dmz004C132] Taxvig C, Hadrup N, Boberg J, Axelstad M, Bossi R, Bonefeld-Jorgensen EC, Vinggaard AM In vitro-in vivo correlations for endocrine activity of a mixture of currently used pesticides. Toxicol Appl Pharmacol 2013;272:757–766. 23954766 10.1016/j.taap.2013.07.028

[dmz004C133] Teeguarden JG, Twaddle NC, Churchwell MI, Doerge DR Urine and serum biomonitoring of exposure to environmental estrogens I: bisphenol A in pregnant women. Food Chem Toxicol 2016;92:129–142. 27038865 10.1016/j.fct.2016.03.023

[dmz004C134] Tertti K, Toppari J, Virtanen HE, Sadov S, Ronnemaa T Metformin treatment does not affect testicular size in offspring born to mothers with gestational diabetes. Rev Diabet Stud 2016;13:59–65. 26859658 10.1900/RDS.2016.13.59 PMC5291182

[dmz004C135] Toppari J, Larsen JC, Christiansen P, Giwercman A, Grandjean P, Guillette LJ Jr, Jegou B, Jensen TK, Jouannet P, Keiding N et al Male reproductive health and environmental xenoestrogens. Environ Health Perspect 1996;104:741–803. 8880001 10.1289/ehp.96104s4741 PMC1469672

[dmz004C136] van den Driesche S, Kilcoyne KR, Wagner I, Rebourcet D, Boyle A, Mitchell R, McKinnell C, Macpherson S, Donat R, Shukla CJ et al Experimentally induced testicular dysgenesis syndrome originates in the masculinization programming window. JCI Insight 2017;2:e91204. 28352662 10.1172/jci.insight.91204 PMC5358493

[dmz004C137] van den Driesche S, Macdonald J, Anderson RA, Johnston ZC, Chetty T, Smith LB, McKinnell C, Dean A, Homer NZ, Jorgensen A et al Prolonged exposure to acetaminophen reduces testosterone production by the human fetal testis in a xenograft model. Sci Transl Med 2015a;7:288ra280. 10.1126/scitranslmed.aaa4097 PMC5044981 25995226

[dmz004C138] van den Driesche S, McKinnell C, Calarrao A, Kennedy L, Hutchison GR, Hrabalkova L, Jobling MS, Macpherson S, Anderson RA, Sharpe RM et al Comparative effects of di(n-butyl) phthalate exposure on fetal germ cell development in the rat and in human fetal testis xenografts. Environ Health Perspect 2015b;123:223–230. 25514601 10.1289/ehp.1408248 PMC4348744

[dmz004C139] Vandenberg LN, Chahoud I, Heindel JJ, Padmanabhan V, Paumgartten FJ, Schoenfelder G Urinary, circulating, and tissue biomonitoring studies indicate widespread exposure to bisphenol A. Environ Health Perspect 2010;118:1055–1070. 20338858 10.1289/ehp.0901716 PMC2920080

[dmz004C140] Veroniki AA, Cogo E, Rios P, Straus SE, Finkelstein Y, Kealey R, Reynen E, Soobiah C, Thavorn K, Hutton B et al Comparative safety of anti-epileptic drugs during pregnancy: a systematic review and network meta-analysis of congenital malformations and prenatal outcomes. BMC Med 2017;15:95. 28472982 10.1186/s12916-017-0845-1 PMC5418725

[dmz004C141] Vinggaard AM, Christiansen S, Laier P, Poulsen ME, Breinholt V, Jarfelt K, Jacobsen H, Dalgaard M, Nellemann C, Hass U Perinatal exposure to the fungicide prochloraz feminizes the male rat offspring. Toxicol Sci 2005;85:886–897. 15788727 10.1093/toxsci/kfi150

[dmz004C142] Welsh M, Saunders PT, Fisken M, Scott HM, Hutchison GR, Smith LB, Sharpe RM Identification in rats of a programming window for reproductive tract masculinization, disruption of which leads to hypospadias and cryptorchidism. J Clin Invest 2008;118:1479–1490. 18340380 10.1172/JCI34241 PMC2267017

[dmz004C143] Wilcox AJ, Baird DD, Weinberg CR, Hornsby PP, Herbst AL Fertility in men exposed prenatally to diethylstilbestrol. N Engl J Med 1995;332:1411–1416. 7723797 10.1056/NEJM199505253322104

[dmz004C144] Yao HH, Whoriskey W, Capel B Desert Hedgehog/Patched 1 signaling specifies fetal Leydig cell fate in testis organogenesis. Genes Dev 2002;16:1433–1440. 12050120 10.1101/gad.981202 PMC186321

