# Supplementary material for: Effect of environmental and pharmaceutical exposures on fetal testis development and function: a systematic review of human experimental data
Source: Hum Reprod Update. 2019 Mar 14;25(4):397–421. doi: 10.1093/humupd/dmz004 (PMC6601394; doi:10.1093/humupd/dmz004)
Supplement: HRU-18-0051-R1-SuppTables_dmz004 [file hru-18-0051-r1-supptables_dmz004.doc]

|  |  |  |  |
| --- | --- | --- | --- |
|  | **Search** | **Search Term** | **Number of hits** |
|  | #1 | Search ‘endocrine disruptor’ OR ‘endocrine disruption’ | 9951 |
|  | #2 | Search ‘human’ AND ‘fetal’ AND ‘testis’ | 2241 |
| **EMBASE** | #3 | #1 AND #2 | **102** |
|  | #4 | Search ‘testosterone’ AND #2 | **321** |
|  | #5 | Search ‘germ cells’ AND #2 | **280** |
|  | #6 | Search ‘Sertoli’ AND #2 | **339** |
|  | #7 | Search ‘Leydig’ AND #2 | **349** |
|  |  |  |  |
|  | **Search** | **Search Term** | **Number of hits** |
|  | #1 | Search ‘endocrine disruptor’ OR ‘endocrine disruption’ | 13810 |
|  | #2 | Search ‘human’ AND ‘fetal’ AND ‘testis’ | 2241 |
| **Pubmed** | #3 | #1 AND #2 | **113** |
|  | #4 | Search ‘testosterone’ AND #2 | **413** |
|  | #5 | Search ‘germ cells’ AND #2 | **555** |
|  | #6 | Search ‘Sertoli’ AND #2 | **350** |
|  | #7 | Search ‘Leydig’ AND #2 | **407** |

**Supplementary Table 1**: Search terms (conducted 13/07/18) and number of hits identified. Records screened shown in Bold.

|  | **Exposure** | **Study** |
| --- | --- | --- |
| **Environmental** | Phthalates | Hallmark 2007 |
|  |  | Lambrot 2009 |
|  |  | Muczynski 2012a |
|  |  | Muczynski 2012b |
|  |  | Heger 2012 |
|  |  | Mitchell 2012 |
|  |  | Desdoits-Lethimonier 2012 |
|  |  | Spade 2014 |
|  |  | van den Driesche 2015 |
|  | Bisphenols | Ben Maamar 2015 |
|  |  | Eladak 2015 |
|  |  | Eladak 2018 |
|  | Pesticides | Bendsen 2001 |
|  |  | Fowler 2007 |
| **Pharmaceutical** | Analgesics | Mazaud-Guittot 2013 |
|  |  | van den Driesche 2015 |
|  |  | Ben Maamar 2017 |
|  |  | Hurtado-Gonzalez 2018 |
|  | DES | N'Tumba-Byn 2012 |
|  |  | Mitchell 2013 |
|  | Metformin | Tartarin 2012 |
| **Lifestyle** | Smoking | Coutts 2007 |
|  |  | Fowler 2009 |
|  |  | Angenard 2010 |
| **Various** | Single agent/mixtures | Gaudriault 2017 |

**Supplementary Table 2**: Publications involving experimental studies on exposure(s) effects using human fetal tissue or cells.

| **Study** | **Reason for Exclusion** |
| --- | --- |
| Johnson 2012 | Review article |
| Albert 2014 |
| Rouiller-Fabre 2014 |
| Habert 2014a |
| Habert 2014b |
| Jegou 2015 |
| Hurtado- Gonzalez 2017 |
| Kilcoyne 2017 |
| Smals 1991 | Data based on exposure during adulthood |
| Xiao 2014 |
| Kristensen 2018 |
| Kellokompu-Lehtinen 1991 | Data based on in-vivo exposure |
| Taxvig 2013 | Data based on human adrenal cell line |
| Rouiller-Fabre 2008 | Article in French |
| Culha 2017 | Comment |

**Supplementary Table 3:** Publications excluded from the search after review of full-text articles and reasons for exclusion

|  | **Exposure** | **Study** |
| --- | --- | --- |
| **Environmental** | Phthalates | Swan 2005 |
|  |  | Main 2006 |
|  |  | Marsee 2006 |
|  |  | Bay 2007 |
|  |  | Swan 2008 |
|  |  | Huang 2009 |
|  |  | Krysiak-Baltyn 2012 |
|  |  | Suzuki 2012 |
|  |  | Bornehag 2015 |
|  |  | Chevalier 2015 |
|  |  | Fenichel 2015 |
|  |  | Swan 2015 |
|  |  | Jensen 2016 |
|  |  | Martino-Andrade 2016 |
|  | Bisphenols | Miao 2011 |
|  |  | Chevrier 2012 |
|  |  | Fenichel 2012 |
|  |  | Chevalier 2015 |
|  |  | Komarowska 2015 |
|  |  | Fernandez 2016 |
|  | Pesticides | Damgaard 2006 |
|  |  | Shen 2008 |
| **Pharmaceutical** | Analgesics | Berkowitz and Lapinski 1996 |
|  |  | Jensen 2010 |
|  |  | Kristensen 2011 |
|  |  | Philippat 2011 |
|  |  | Snijder 2012 |
|  |  | Fisher 2016 |
|  |  | Lind 2017 |
|  | DES | Henderson 1976 |
|  |  | Gill 1979 |
|  |  | Leary 1984 |
|  |  | Martin 2008* |
|  |  | Palmer 2009 |
|  | Antifungals | Carter 2008 |
|  |  | Norgaard 2008 |
|  |  | Mogensen 2017 |
| **Lifestyle** | Alcohol | Damgaard 2006 |
|  | Smoking | Storgaard 2003 |
|  |  | Ramlau-Hansen 2007 |
|  |  | Jensen 2007 |
|  |  | Damgaard 2008 |
|  |  | Fowler 2009 |
|  |  | Fowler 2011 |

**Supplementary Table 4**: Epidemiological studies for associations between in-utero exposures and TDS disorders. * Meta-analysis of all available studies
